# Comparison of the Prognostic Utility of the Diverse Molecular Data among lncRNA, DNA Methylation, microRNA, and mRNA across Five Human Cancers

**DOI:** 10.1371/journal.pone.0142433

**Published:** 2015-11-25

**Authors:** Li Xu, Liang Fengji, Liu Changning, Zhang Liangcai, Li Yinghui, Li Yu, Chen Shanguang, Xiong Jianghui

**Affiliations:** 1 School of life science and biotechnology, Harbin Institute of Technology, Harbin, Hei Longjiang Province, China; 2 State Key Laboratory of Space Medicine Fundamentals and Application, Space Institute of Southern China, China Astronaut Research and Training Center, Beijing, China; 3 National Key Laboratory of Human Factors Engineering, China Astronaut Research and Training Center, Beijing, China; 4 Xishuangbanna Tropical Botanical Garden, Chinese Academy of Sciences, Yunnan, China; 5 Department of statistics, Rice University, Houston, Texas, United States of America; Ospedale Pediatrico Bambino Gesu', ITALY

## Abstract

**Introduction:**

Advances in high-throughput technologies have generated diverse informative molecular markers for cancer outcome prediction. Long non-coding RNA (lncRNA) and DNA methylation as new classes of promising markers are emerging as key molecules in human cancers; however, the prognostic utility of such diverse molecular data remains to be explored.

**Materials and Methods:**

We proposed a computational pipeline (IDFO) to predict patient survival by identifying prognosis-related biomarkers using multi-type molecular data (mRNA, microRNA, DNA methylation, and lncRNA) from 3198 samples of five cancer types. We assessed the predictive performance of both single molecular data and integrated multi-type molecular data in patient survival stratification, and compared their relative importance in each type of cancer, respectively. Survival analysis using multivariate Cox regression was performed to investigate the impact of the IDFO-identified markers and traditional variables on clinical outcome.

**Results:**

Using the IDFO approach, we obtained good predictive performance of the molecular datasets (bootstrap accuracy: 0.71–0.97) in five cancer types. Impressively, lncRNA was identified as the best prognostic predictor in the validated cohorts of four cancer types, followed by DNA methylation, mRNA, and then microRNA. We found the incorporating of multi-type molecular data showed similar predictive power to single-type molecular data, but with the exception of the lncRNA + DNA methylation combinations in two cancers. Survival analysis of proportional hazard models confirmed a high robustness for lncRNA and DNA methylation as prognosis factors independent of traditional clinical variables.

**Conclusion:**

Our study provides insight into systematically understanding the prognostic performance of diverse molecular data in both single and aggregate patterns, which may have specific reference to subsequent related studies.

## Introduction

Cancer prognosis prediction is crucial to controlling the suffering, progression, and death of patients. Accurate outcome prediction can be used clinically to select the best of several available therapies for cancer patients and improve their chances of survival[1, 2]. Traditionally, prognosis is based on clinical pathological parameters such as tumor stage, metastasis, and pathologic diagnostic age[3]. Recently, a number of distinctive molecular biomarkers have been surveyed and applied to access the clinical outcome of patients, such as protein-based (phosphorylation states, cell surface receptors), DNA-based (SNP, CNV), and the RNA-based (mRNA, microRNA, ncRNA) [4–7]. Additionally, there is growing evidence suggesting that long non-coding RNA (lncRNA) and DNA methylation can mediate oncogenic or tumor suppressive outcomes, representing new classes of promising biomarkers[5]. However, most studies focus on either one single cancer lineage or on individual platform data, whereas a comprehensive comparison to determine the relative prognostic power for each class of molecules for a specific cancer would ideally provide a more effective diagnostic platform. This would also allow consideration of whether targeting the joint biomarkers would provide better control of cancers[2]. Despite the growing availability of data describing these various molecules, previous studies or available frameworks/pipelines have not investigated these questions.

Strategies such as RT-PCR and immunohistochemistry have investigated a considerable number of biomarkers for prognosis[8–10]. However, most of the biomarkers were found by “educated guesses” rather than *via* a systematic, genome-wide approach. Additionally, only a few have been used in a clinical setting and the utility of the majority of these wet-lab-based markers remains to be determined[4]. More recently, using high-throughput profiles, computational approaches like machine-learning approaches [11–16] and different survival models [17–19] are being applied to identify candidate biomarkers with prognostic values for disease. While these methodologies have accumulated large amount of molecular signatures with acceptable accuracies, little systematic research has been performed to determine the prognostic power of diverse molecular signatures and their relative importance. This is because most studies suffer from one or several of the following four problems: (i) deficiency of molecular profiles, (ii) limited to single cancer lineage, (iii) underdeveloped strategies to explore optimal predictors in terms of high dimension data and tumor heterogeneity. Nevertheless, The Cancer Genome Atlas (TCGA) project aggregated large quantity of genomic data was found to increase the understanding the clinical pathologies of different molecular platforms in human cancers[20–23], which would help the translation of biological data into prognostic utility.

In this study, we have implemented a pipeline to identify prognosis-related biomarkers in multi-omic profiles including RNA-seq, DNA methylation Bead ChIP, and microRNA-seq and compared their relative prognostic power in five TCGA cancers. During the modeling process, biomarkers crucial to clinical outcome were ranked and selected using our Iterative Deletion Feature Optimal (IDFO) approach. Moreover, we assessed the predictive utilities of both individual and integrated multi-omic predictors to investigate their contribution to model performance, and the predictive power of diverse molecular predictors in respective cancers were further evaluated in independent test sets. Survival analysis was used to determine the prognostic utility of IDFO-identified predictors alone or in combination with clinical variables. Furthermore, to facilitate the use of our approach, we also implemented a publicly available R source code (CAPM.R), which allows researchers to build prognosis models for other datasets. Our study provides a dynamic risk assessment system for cancer prognosis prediction, which not only reveals the prognostic utility of multi-omic data across cancer types, but also facilitates the understanding of lncRNA and DNA methylation as potential prognostic markers on tumor progression.

## Materials and Methods

### Datasets

We assembled 3198 publicly available tumor samples into array-based data among five types of cancers from The Cancer Genome Atlas (TCGA) project, which have been published in [20, 24–27] (Table A in S1 File describes the detailed sample distributions). All tumor samples were selected based on the following criteria: (a) signatures (mRNA/lncRNA/microRNA transcripts, DNA methylation probes) absent in 50% of the tumor samples were removed as the irrelevant, (b) samples with matched clinical information (e.g., survival time, age, tumor stage), (c) tumor patients with only up to one month survival after surgery were excluded to avoid any potential confounding influence of postoperative complications. Most of the tumor samples were composed of three different molecular profiling data sets, which were RNA-seq, microRNA-seq, and DNA methylation Bead ChIP. Four types of molecular signatures were extracted as prognosis predictors from the three molecular data profiles, including lncRNA and mRNA signatures from RNA-seq profiles, DNA methylation signatures were from the DNA methylation Bead ChIP 450k/27k, and microRNA signatures were from the microRNA-seq profiles. For each molecular data profile, we randomly selected two-third of tumor samples to construct (i.e. ‘train’) prediction models to identify best performance predictors, and the remaining third of samples were utilized for an independent test of these predictors. Datasets corresponding to different cancers were analyzed separately. Moreover, for predicting the outcome of patients, tumor samples were assigned to either a ‘good’ or ‘poor’ outcome groups as prognosis labels. The threshold of two outcome groups was defined on the basis of clinical characterization of respective cancers (which have the advantage of yielding two outcome groups with equal size in each cancer).

### Dichotomization of survival data

We dichotomized the censored survival data for each type of cancer by assigning a threshold of cutoff time as: 2 years for patients with colon adenocarcinoma (COAD), 3 years for lung squamous cell carcinoma (LUSC), serous cystadeno carcinoma (OV), uterine corpus endometrioid carcinoma (UCEC), and 5 years for breast invasive carcinoma (BRCA). The patients who lived beyond the cutoff time were labeled as ‘good prognosis’ the deceased were labeled as ‘bad prognosis’. Patients with censored survival times that were before the cutoff threshold were excluded (e.g., less than 1 month).

### Pre-processing of genomic and epigenome profiles

RNA-seq: TCGA RNA-seq level 2 data were normalized and processed by calculating the reads per kilo base per million mapped reads (RPKM) value for the expression of lncRNA/mRNA transcripts. To match the assembled transcripts into detailed lncRNAs/mRNAs, all transcripts were aligned to the Human Genome by the reference list from the UCSC (GRCh37/hg19), while transcripts with > half of its lengths with in an lncRNA/mRNA were identified as a match[28].

MicroRNA-seq: microRNA expression levels were assayed *via* TCGA microRNA sequencing level 3 data (Illumina Genome Analyzer & Hiseq 2000). The calculated expressions for transcripts aligning to a particular miRNA were retrieved from both the miRNA isoform and quantification files (available at the TCGA data portal along with metafiles annotating each dataset)[29].

DNA Methylation Bead ChIP: The DNA methylation data sets in most tumor cohorts are composed of the Illumina 450K and/or 27K array platforms. Accordingly, we selected the overlaps CpGs (measured with the Infinium type II assay) that were present on both of the two platforms (Infinium 450K and 27K) and had no more than 10% missing values across all samples in each type of cancer, respectively.

### Signature evaluation methodology: IDFO

**Fig 1:**
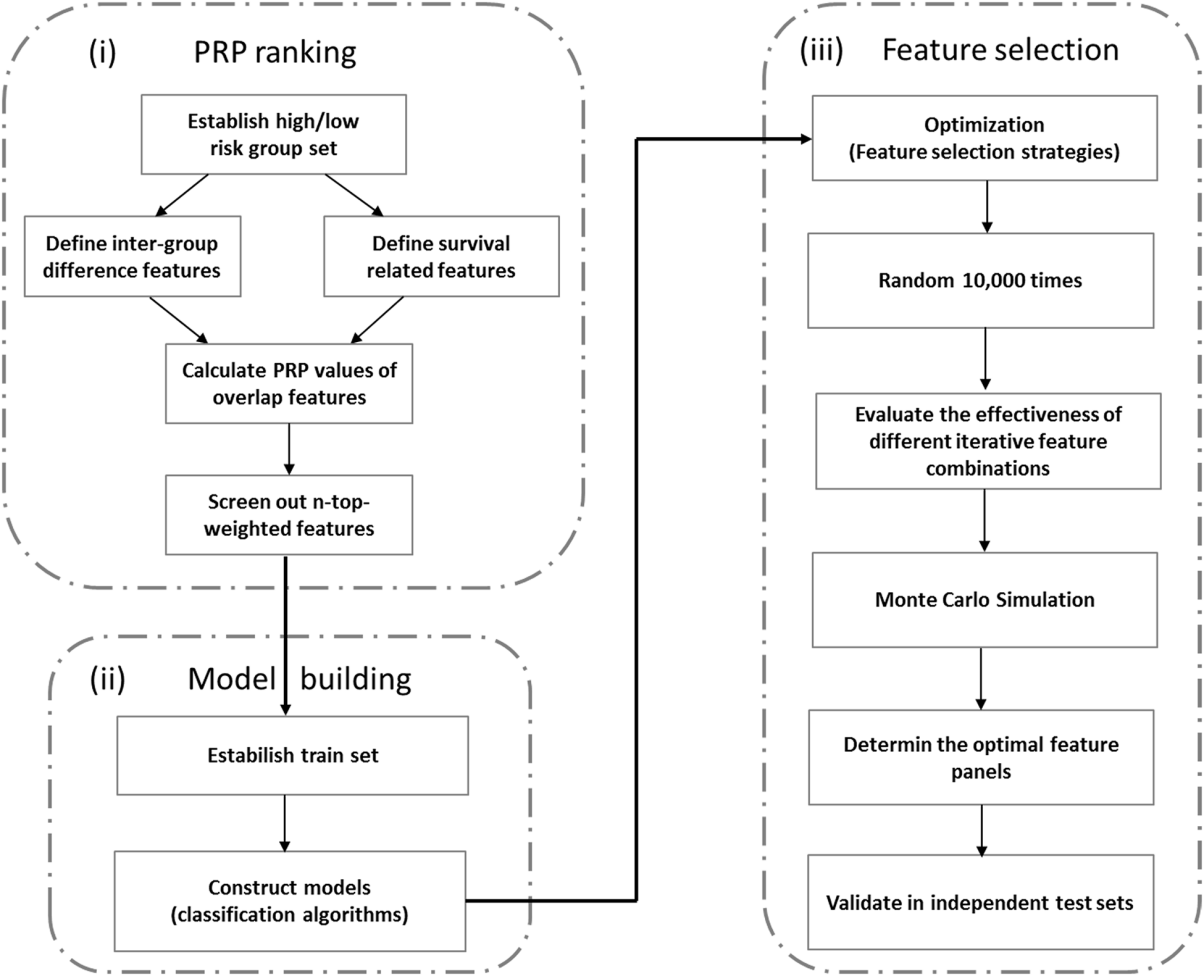
Flowchart of the IDFO approach. This flowchart contains three basic steps: (i) PRP ranking of molecular features, (ii) model construction and (iii) feature optimization and validation.

The IDFO approach was composed of three basic procedures (Fig 1):

1. The Prognosis Risk Prioritization (PRP) ranking. There were a large number of candidate variables within the diverse molecular profiles, which would cost enormous calculation during model training. To overcome this “dimension curse”, we developed this pre-biomarker ranking strategy: Prognosis Risk Prioritization (PRP) to screen out the most representative prognostic variables as initially model training features for each molecular profile, respectively. In this process, we explored two steps:
  - a calculation of $Z_{x_{i}}$ for extracting differentially expressed/methylated signatures *x*_*i*_ between the two outcome groups. As formula,
    $$Z_{x_{i}}=\frac{\bar{G_{1}}-\bar{G_{2}}}{\sqrt{\frac{1}{2}\left(\sigma_{1}^{2}+\sigma_{2}^{2}\right)}}$$ (1)
    Here $\bar{G_{1}}$ was the average expression/methylation value of signature *x*_*i*_ in the 1^st^ group, and $\bar{G_{2}}$ was the average expression/methylation value of *x*_*i*_ in the 2^ed^ group, *σ* was the standard deviation of two respective group, 1 = group one, 2 = group two.
  - *P*_*unicox*_, a calculation of univariate Cox *p* value of molecular signature *x*_*i*_, which used the expression/methylation values of *x*_*i*_ as the variable for a univariate Cox regression survival analysis.
    Finally, the *PRP*_*risk value*_ of signature *x*_*i*_ was calculated as using this formula,
    $$PRP_{risk\;value}=-Z_{x_{i}}{\text{log}}_{10}\left(P_{unicox}\right)$$ (2)
    Where $Z_{x_{i}}$ derived from Eq 1.
2. Model building. For comprehensively evaluating the prognostic ability of multi-platform molecules to respective cancer types, we utilized 5 machine learning models in combination with 4 feature extraction strategies to establish a performance pipeline. Two other steps were used: model building and feature selection. Five machine learning algorithms (see in Supplementary Methods in S1 File) were proposed in model building, which are support vector machine (SVM), k-nearest neighbors (KNN), logistic regression (LR), random forest (RF) and NaiveBayes (NB). The performance of each classifier was evaluated using 632—Bootstrap method, using this formula,
  $${\text{Boot}}_{acc}=\frac{1}{n}\overset{n}{\underset{i=1}{\sum }}(0.368\times acc_{train}^{i}+0.632\times acc_{test}^{i})$$ (3)
  Where *n* was the total number of repeats, $acc_{train}^{i}$ and $acc_{test}^{i}$ were the *i*_*th*_ experiment train accuracy and test accuracy. Here we split two-thirds samples for training and one-thirds samples for testing, both of which were extracted from the original training sets.
3. Feature selection. In this procedure, we proposed four feature extraction strategies, namely as SVM-RFE, RF-IS, LASSO and PFS (Supplementary Methods in S1 File) to determine the optimal set of features comprehensively. The feature selection procedure started with the PRP algorithm ranked n-top-weighted features (for detailed numbers see Supplementary Methods and Figure A in S1 File) and then iteratively eliminated a number or a fraction of the least important/crucial features determined based on respective extraction strategies until the highest bootstrap accuracy was obtained. During the feature optimization process, an average accuracy of 10,000 times random re-sampling with replacement was calculated as the estimate accuracy for each iterative selected feature sets. To evaluate the stability of the PRP feature ranks, a Monte Carlo simulation using R package GMCT[30] was also performed by randomly selecting equal number features for the respective molecular models in each tumor. Finally, the highest bootstrap model was identified as the best prognosis model and its screened out features were then tested in test set for independent validation. The model construction, statistical analysis and graphs were performed using Bioconductor (www.bioconductor.org).

### Statistics

Student’s *t* tests were used to compare differential expressed signatures (mRNAs, lncRNAs, DNA methylation, and microRNAs) among two risk groups. The Wilcoxon signed rank test was adopted to assess the statistical significance of survival distribution of the two prognosis groups classified by MCPHR models, per this formula,

$${\text{χ}}^{2}=\frac{{\left(S_{1}+S_{2}\right)}^{2}}{\left(V_{1}+V_{2}\right)}$$ (4)

Where *S*_1_ and *S*_2_ was the rate of survivors in two risk groups, *V*_1_ and *V*_2_ was the variance of *S*_1_ and *S*_2_.

The likelihoods ratio test was used to compare the fit of two models (e.g., IDFO predictor models with and without additional variables) which was based on computing the likelihood ratios. The likelihood function was calculated using this formula,

$$L(\beta )=\prod_{i=1}^{n}{\left[f(t_{i})\right]}^{w_{i}}{\left[S(t_{i})\right]}^{1-w_{i}}$$ (5)

Where *S*(*t*_*i*_) was the survival function which showed the proportion of the *i*_*th*_ patient alive at time *t*; The density function *f*(*t*_*i*_) was the probability of dying in the small interval of time *t*; *w*_*i*_ was the weighted mean survival rate calculated from 1Vi, where *V*_*i*_ was the variance of survival rates; *n* was the total number of patients in respective cohorts. The Kaplan–Meier Analysis and log-rank likelihood models were used to test for differences in survival and the Kaplan-Meier curves were drawn based on the median risk score. The *p* values in all statistical tests less than 0.05 were considered significant. The above statistical analyses were performed using the R packages: ‘survival’ and ‘survcomp’.

### Multivariate Cox proportional Hazard Regression

The multivariate Cox proportional hazard regression model, as the most popular mathematical modeling approach was applied to estimate the hazard ratios, relative risks, corresponding 95% confidence intervals (CI) and survival curves by using several/multiple explanatory variables (molecular and/or clinical variables). As an example, a parametric model was based on the exponential distribution using this formula,

$$\text{log}h(t)\;=\alpha +\beta_{1}x_{1}\;+\beta_{2}x_{2}+・・・+\beta_{k}x_{k}$$ (6)

Where, *h*(*t*) was the hazard function, the *x*_1_,*x*_2_,*…x*_*k*_ were the covariates, and *β*_1_,*β*_2_,*…*,*β*_*k*_ were the coefficients of respective covariates, where, *β >0* represented the covariate risk factor related with ‘poor prognosis’ on the contrary, *β <0* indicated the covariate protected factor related with ‘good prognosis’. The constant *α* in this model represented a log-baseline hazard, since log *h*(*t*) = *α* or *h*(*t*) = *exp*(*α*)when all of the *x* values were zero.

*Risk scores*. *R*(*t*) was computed for the prognostic risk of each patient, and defined as a linear combination of predictor variables weighted by their respective Cox regression coefficients, and calculated using this formula,

$$R(t)=exp^{-h\left(t\right)}$$ (7)

Where *R*(*t*) was the risk score of patient *t*, *h*(*t*) was the hazard value calculated by the multivariate Cox regression model (derived by Eq 6).

### R codes: CAPM

To allow users to apply our constructed pipeline to other data sets, we implemented a publicly available R source code (CAPM.r) to perform cancer prognosis prediction, which is freely available at http://www.escience.cn/people/lixu/index.html.

## Results

### Evaluation of the prognostic performance of diverse molecular data

**Fig 2:**
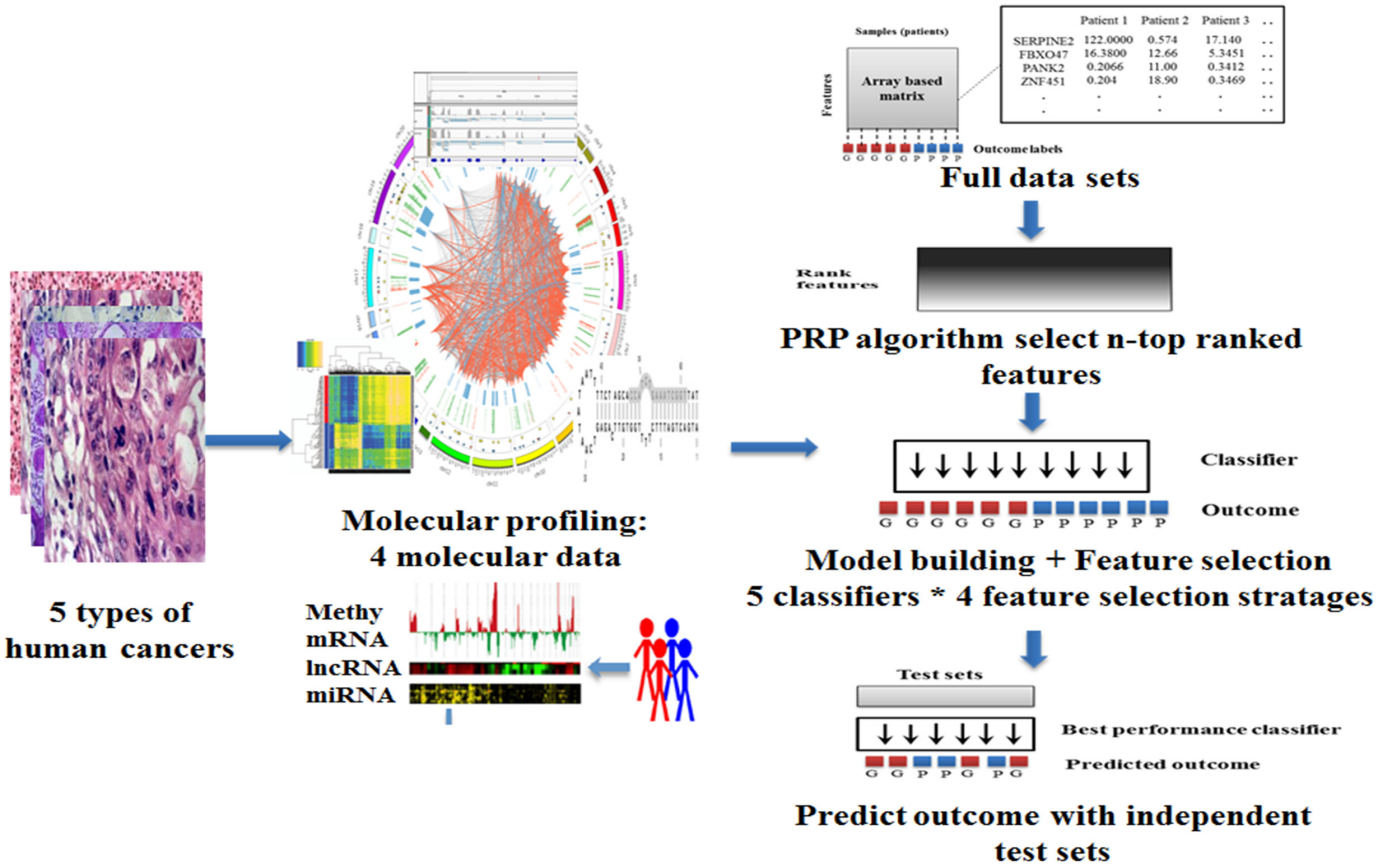
An overall scheme of the prediction pipeline. This pipeline contains four mainly procedures: I) Data processing. We assembled a collection of 3198 tumor samples in five types of human cancers, which composed of four types of molecular data including: lncRNA, microRNA, mRNA, and DNA methylation. Each type of molecular data in respective cancers was processed into array based matrix using CAPM preprocessing methods. II) Feature ranking. Molecular features associated with prognosis were analyzed and sorted according to the PRP algorithm. For each type of molecular data, we selected top-weighted 100 signatures as the initial feature sets in each of the five cancers, respectively. III) Model building and feature selection. In this process, we adopted five classifiers in combination with four feature selection algorithms to establish the prognosis prediction baseline. During the feature selection process, each group of features was trained with 10,000 times randomization and the best performing feature panel with highest bootstrap accuracy was chosen for each molecular data per cancer. IV) Validation. To evaluate the predictive power of each molecular data, the best performing features were further applied to independent test in each cancer cohorts, respectively.

The flow chart of our study is shown in Fig 2. We assembled 3198 publicly available tumor samples into array-based data among five TCGA cancer types: breast invasive carcinoma (BRCA)[26], colon adenocarcinoma (COAD)[27], lung squamous cell carcinoma (LUSC)[25], uterine corpus endometrioid carcinoma (UCEC)[31] and serous cystadeno carcinoma (OV)[24]. The five cancer types were chosen because their TCGA cohorts included sufficient samples with multiple types of molecular data and clinical information (Table A in S1 File). Each cancer type was composed of four molecular data profiles, including (i) lncRNA: Illumina HiSeq 2000 RNA Sequencing V2; (ii) mRNA: Illumina HiSeq 2000 RNA Sequencing V2; (iii) DNA methylation: Illumina Infinium Human DNA Methylation 27K, 450k; (iv): microRNA: Illumina Genome Analyzer/HiSeq 2000 microRNA sequencing platform. In order to comprehensively evaluate the predictive power of the four types of molecular signatures to their respective cancers, we constructed a group of 5 classifiers (SVM[32], KNN[33], NaiveBayes[34], RandomForest[35], Multinomial logistic regression[14]) in combination with 4 feature extraction strategies: The Least Absolute Shrinkage and Selection Operator (LASSO)[36], Support Vector Machine based Recursive Feature Elimination (SVM-RFE)[37], Random Forest importance spectrum based feature selection (RF-IS)[38], and Prioritization-eliminated feature selection (PFS) (Supplementary Methods in S1 File) to build a prognosis computational pipeline which named as the Iterative Deletion Feature Optimization method (IDFO, see Methods and Fig 1).

**Fig 3:**
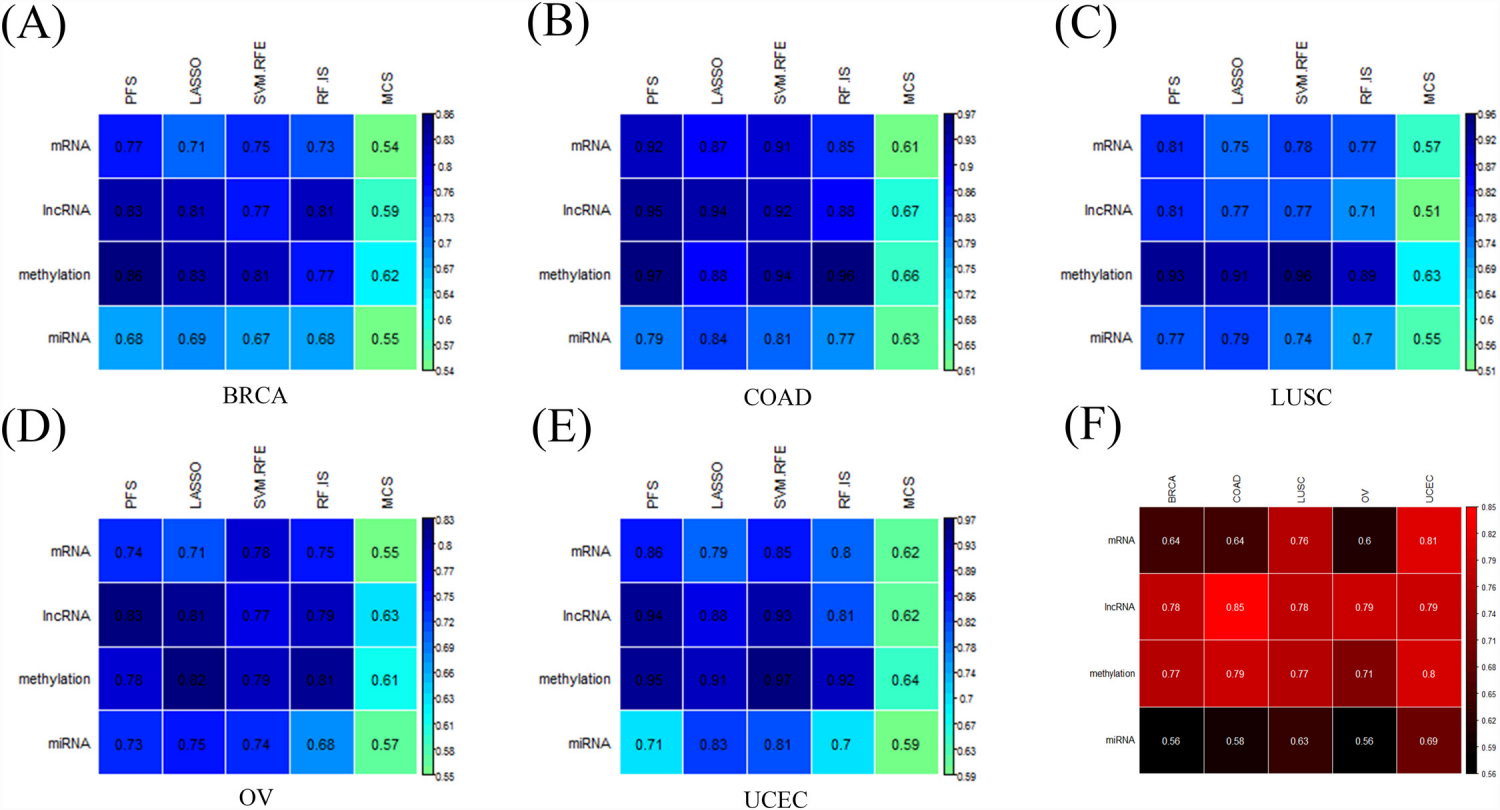
The performances of diverse molecular signatures in training (A-E) and testing (F) data sets across five TCGA cancers. (A-E) Best predictive models of each molecular data of five human cancers across different feature selection strategies (columns indicated feature selection strategies: LASSO, PFS, SVM-RFE, RF-IS, and MCS; rows indicated molecular signatures), (F) Test set accuracies of four types of molecular signatures in five TCGA cancers (rows indicated molecular data types, columns indicated cancer types). In order to distinguish the results between training and testing, we utilized blue-colored items for training results (Fig 3A–3E) and red-colored items for testing results. * BRCA = breast invasive carcinoma; COAD = colon adenocarcinoma; LUSC = Lung squamous cell carcinoma; UCEC = Uterine Corpus Endometrioid Carcinoma; OV = Serous cystadenocarcinoma.

During the feature optimization process, the classifiers for each molecular data were initially trained by the Prognosis Risk Prioritization algorithm (PRP; see Methods) ranked features and then iteratively eliminated a number or a fraction of the least important/crucial features which were determined by four-feature extraction strategies until the optimal panel of features was observed. To evaluate the stability of the PRP method, a Monte Carlo Simulation (MCS) was also performed to select equal size of features as random validation for the respective profiles in each tumor. A classifier with the highest bootstrap accuracy[39] was identified as the optimal model and the best performance predictors were then tested in independent test cohorts. The model performances of each molecular data (‘train’) in combination with respective feature selection strategies are highlighted in Fig 3a–3e. We observed that, 1) the bootstrap accuracies of all classifiers ranged from 0.71 to 0.97 (Table B in S1 File), which indicated good performance of IDFO approach for multiple cancer types; 2) the PRP ranked feature sets resulted in significantly improved accuracy compared with random selected MCS feature sets (average accuracy: PRP = 0.81, MCS = 0.59; one-sided Wilcoxon signed rank test: *P*<1.12e-5); 3), and there was no apparent difference between the classification algorithms with respect to tumors, and the performance of diverse molecular signatures did not vary significantly across cancers, confirming a highly robust of genomic and epigenetic data in prognosis prediction; 4) of all 20 optimal prognostic models (5 cancers * 4 molecular data sets), 12 out of 20 (60%) were obtained by the PFS algorithm, followed by LASSO (30%) and SVM-RFE (10%), which indicated that our novel feature selection approach proposed had good performance similar to traditional methods (Table B in S1 File). Subsequently, to compare the predictive performance of the four types of molecular signatures with an unbiased validation, we applied the best prognosis predictors from each training model to an independent test set. Notably, as is shown in Fig 3f, the lncRNA signatures illustrated the best performance in four cancers: BRCA (test set accuracy: 0.78, *N*_*test set*_ = 159), COAD (test set accuracy: 0.85, *N*_*test set*_ = 48), LUSC (test set accuracy: 0.77, *N*_*test set*_ = 56), and OV (test set accuracy: 0.79, *N*_*test set*_ = 75). DNA methylation was the second best predictor of BRCA (test set accuracy: 0.76, *N*_*test set*_ = 73), COAD (test set accuracy: 0.79, *N*_*test set*_ = 67), LUSC (test set accuracy: 0.77, *N*_*test set*_ = 42), ovarian cancer (test set accuracy: 0.7, *N*_*test set*_ = 146), and the third best predictors in UCEC (test set accuracy: 0.8, *N*_*test set*_ = 81). mRNA and microRNA as traditional clinical baseline markers, were ranked lower than our initial expectations. mRNA was the third best predictors in BRCA (test set accuracy: 0.64, *N*_*test set*_ = 159), COAD (test set accuracy: 0.64, *N*_*test set*_ = 48), LUSC (test set accuracy: 0.76, *N*_*test set*_ = 56) and OV (test set accuracy: 0.6, *N*_*test set*_ = 75). MicroRNA data resulted in worse predictive power compared with all other data types. In addition, due to the remarkable performance of lncRNAs in patient survival stratification, we further performed literature retrieval to examine the possibility for any evidence of the correlation between IDFO-screened lncRNAs and prognosis progression. Of all 157 optimal lncRNA predictors in five cancers (21 in BRCA, 36 in COAD, 33 in LUSC, 41 in OV, 37 in UCEC), 22 lncRNAs had been previously reported in literature (Table F and Figure B in S1 File). These results suggested that our approach could potentially identify trustable prognosis associated lncRNAs, and we posited newly identified lncRNAs, either in isolation or as composite markers, may be crucial to clinical practice.

### Integration of multi-omic biomarkers to prognosis prediction

**Fig 4:**
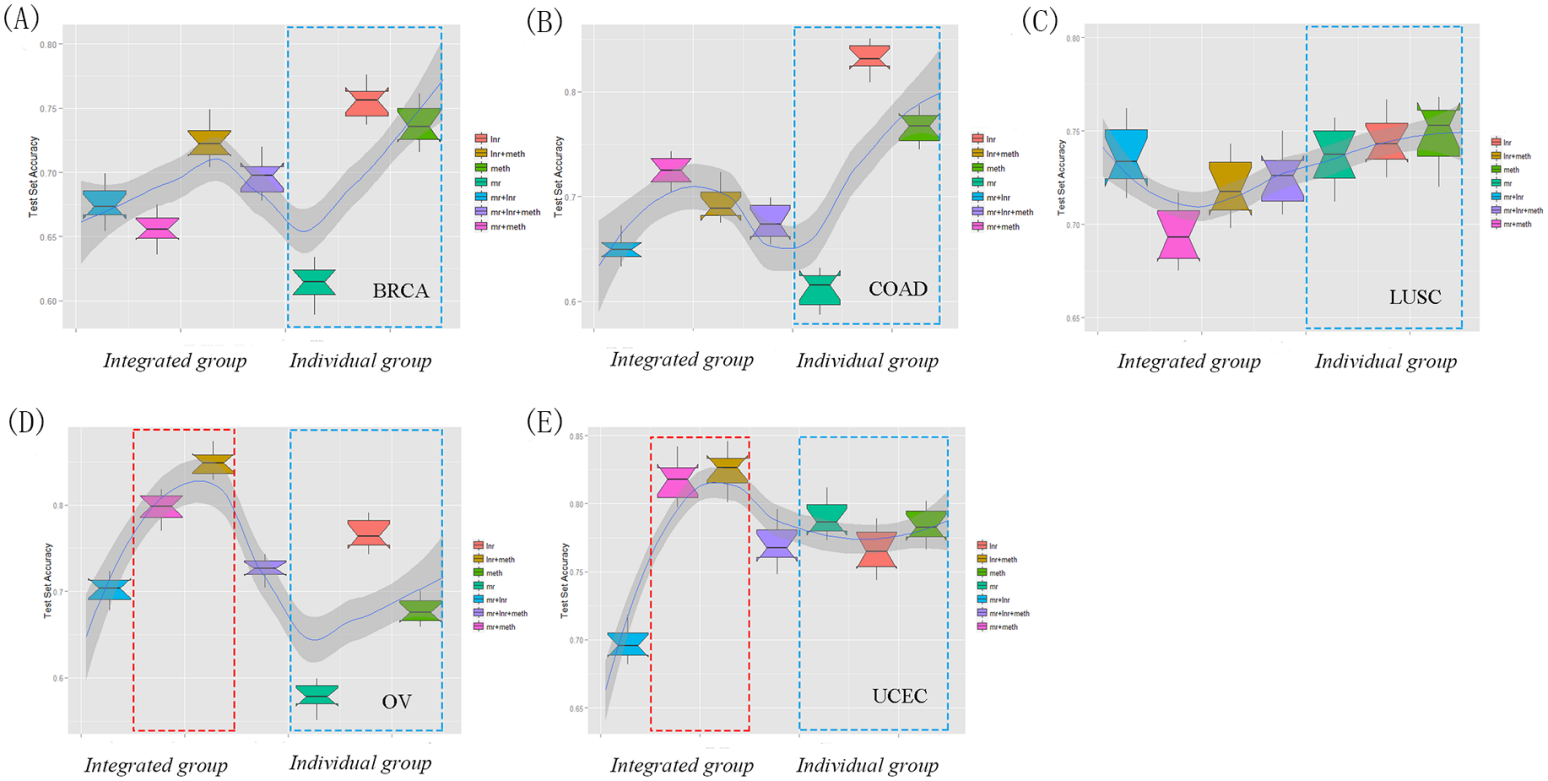
Comparison of the predictive performance of integrated multi-type molecular data and single molecular data in cancer outcome stratification. (A) BRCA (N_*overlaps*_ = 178), (B) COAD (N_*overlaps*_ = 161), (C) LUSC (N_*overlaps*_ = 97), (D) OV (N_*overlaps*_ = 145), (E) UCEC (N_*overlaps*_ = 84). For the respective models in each type of cancer, we performed 10,000 times of random splitting with 2/3 training and 1/3 testing using IDFO pipeline. The dotted red box indicated the significantly improved performance of two integrated models in (D) OV and (E) UCEC compared with individual data type models (two-sided Wilcoxon signed rank test, *P* < 0.01); the dotted blue box indicated the three individual data type models of mr, lnr and meth. The integrated group are composed of both double-combination and triple-combination molecular signature models. Individual group contained the three individual molecular data type models. The gray line across seven boxes shows the predictive patterns of integrated groups and individual groups. N_*overlaps*_ is the number of overlap sample occurred in all three molecular data profiles (mRNA, lncRNA and DNA methylation), lnr = lncRNA, mr = mRNA, meth = DNA methylation, mr+lnr = mRNA + lncRNA, mr+meth = mRNA + DNA methylation, lnr+meth = lncRNA + DNA methylation, mr+lnr+ meth = mRNA + lncRNA +DNA methylation.

Recent studies suggested the integrated multi-omic signatures could efficiently improve the model performance[28, 40]. To explore whether such hypothesis was appropriate to the dichotomized overall survival prediction, we extended our IDFO approach to investigate the performance of integrative modeling of multi-type molecular data in five cancers. As integrative models require samples not only comprised of multi-omic profiles, but also those that fulfill the prognostic criteria, we observed a final of 20 integrated multi-omic data groups in the five cancer types, including 15 double-combination groups and 5 triple-combination groups (see Table C in S1 File). As there were an insufficient number of microRNA-seq samples overlapping with the three other molecular profiles, the microRNA signatures were excluded in the integrated modeling analysis. Table C in S1 File listed the predictive accuracies (‘test’) of the 20 integrated models. In sum, 80% of the integrated multi-omic data combinations did not show significantly improved predictive power compared to their individual molecular data (Fig 4A–4C), except for lncRNA + DNA methylation models in two cancer types of OV and UCEC (Fig 4D and 4E) (OV: one-sided Wilcoxon signed rank test, DNA methy+ lncRNA *vs*. DNA methy: *P* < 1.2e−4, DNA methy+ lncRNA *vs*. lncRNA: *P* <4.7e−3; UCEC: DNA methy+ lncRNA *vs*. DNA methy: *P* <1.7e−4, DNA methy+ lncRNA *vs*. lncRNA: *P* <8.2e−5). Besides, with the increase of molecular types, the performance of triple combination groups was in accordance with the average level of the single-type molecular models with limited perturbations in all five cancer types. Consequently, most of the integrated multi-omic data models showed similar predictive power with their respective individual molecular data models, suggesting the information content of integrated multi-platform data might largely be redundant in terms of patient survival stratification. Similar results were also observed in a recent breast cancer modeling treatment study [1].

### Survival analysis: validation of IDFO predictors on censored survival data

**Fig 5:**
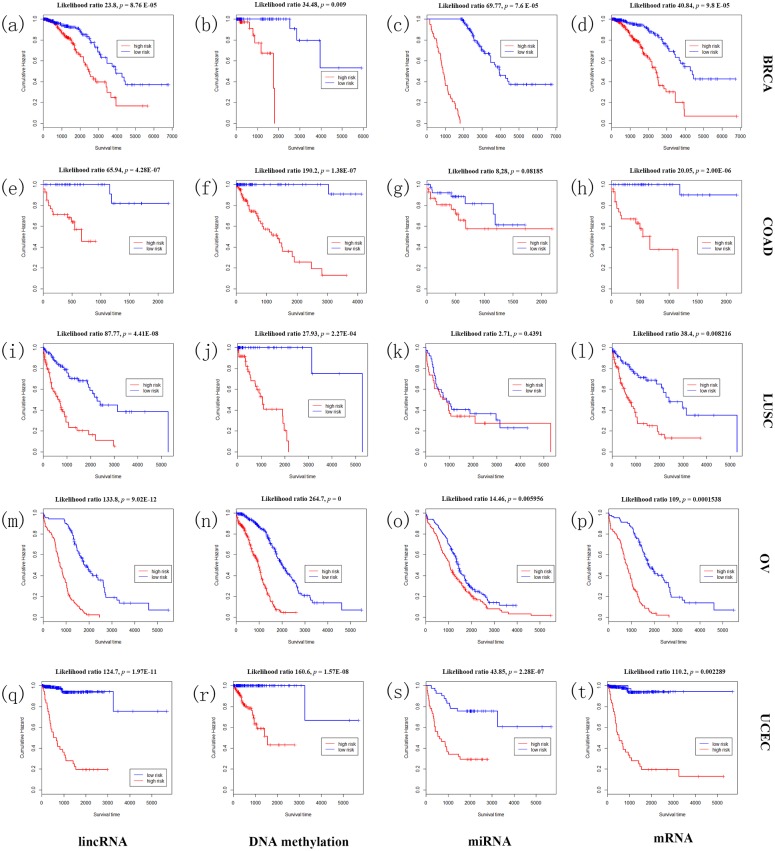
Survival analysis on IDFO predictors of four types of molecular data in five cancers. The Kaplan-Meier overall survival curves of two outcome groups classified by MCPHR models using IDFO-identified predictors of each molecular data of each cancer. (a) the BRCA lncRNA cohort; (b) the BRCA DNA methylation cohort; (c) the BRCA microRNA cohort; (d) the BRCA mRNA cohort; (e) the COAD lncRNA cohort; (f) the COAD DNA methylation cohort; (g) the COAD microRNA cohort; (h) the COAD mRNA cohort; (i) the LUSC lncRNA cohort; (j) the LUSC DNA methylation cohort; (k) the LUSC microRNA cohort; (l) the LUSC mRNA cohort;(m) the OV lncRNA cohort; (n) the OV DNA methylation cohort; (o) the OV microRNA cohort; (p) the OV mRNA cohort;(q) the UCEC lncRNA cohort; (r) the UCEC DNA methylation cohort; (s) the UCEC microRNA cohort; (t) the UCEC mRNA cohort. The difference in outcome of two outcome groups was tested using Kaplan-Meier survival analysis. Likelihood ratio = the likelihood ratio test.

In addition to examine the association between IDFO predictors and clinical outcome in BRCA, COAD, LUSC, UCEC and OV, we subjected the best predictors of respective data profiles to the multivariate Cox proportional hazard regression (MCPHR) analysis[41] to evaluate the correlation of IDFO-predictors with prognosis risk and investigate their clinical utilities. Here, we utilized the MCPHR models to compute the relative risks (RR) of tumor patient and classified the patients into two prognosis groups (‘good prognosis’ and ‘poor prognosis) according to the median risk scores in respective molecular data profiles. As shown in Fig 5, in UCEC (n = 586), the three year survival of lncRNA cohort approached 92% in ‘good prognosis’ and 19% in ‘bad prognosis’ (Chisq = 44.5, *P* = 1.67e-09, log rank test); mRNA cohort approached 91% in ‘good prognosis’ group and 20% in ‘bad prognosis’ group (Chisq = 29.3, *P* < 1e-10, log rank test); while DNA methylation cohort approached 99% in ‘good prognosis’ group and 40% in ‘bad prognosis’ group (Chisq = 17.5, *P* = 0.0073, log rank test); and in microRNA cohort, the three year survival of two risk groups approached 77% and 28% (Chisq = 14.1, *P* = 1.59e-09, log rank test). In BRCA (n = 671), we obtained a five year survival of 92% and 65% in two risk groups of lncRNA cohorts (Chisq = 41.5, *P* = 1.76e-05, log rank test); 89% and 74% in mRNA cohorts (Chisq = 38.2, *P* = 7.3e-05, log rank test); 99% and 68% in DNA methylation cohorts (Chisq = 22.5, *P* = 0.004669, log rank test); and 100% and 16% in microRNA cohorts (Chisq = 18.4, *P* = 0.008759, log rank test). Similar results of statistically significances were also observed in COAD, LUSC, and OV (details in Table D in S1 File). Notably, most IDFO predictors emerged as significant variables related to survival (log rank *p* < 0.01, Table D in S1 File), and the classified two risk groups in respective molecular cohorts were associated with statistically significant differences in overall survival (OS) except for the microRNA cohort in LUSC (logrank test, *p* = 0.4014, Fig 5k), which suggested a compelling advantage of IDFO predictors in both dichotomized and/or censored survival prediction.

Many studies have suggested that clinical variables provide additional predictive power to prognosis models [3, 4, 42]. Therefore, we extended the MCPHR model of each type of molecular data with four additional clinical variables (a) tumor stage, (b) tumor grade, (c) pathologic diagnostic age, and (d) sex in order to test whether clinical factors would improve the prognosis predictions in combination with molecular signatures. We compared the performance (C- index[43], see Supplementary Methods in S1 File) of each molecular prognostic model with and without clinical variables by computing the *P* values of two-sided Wilcoxon signed rank test (see Methods) in each cancer type, respectively. Interestingly, the molecular data + clinical models resulted in improved predictive performance compared with single molecular data models in most cancer types, especially in the mRNA and microRNA cohorts (Table E in S1 File). For example, the microRNA + clinical models in BRCA (two-sided Wilcoxon signed rank test: *P*< 2.1e-3), LUSC (two-sided Wilcoxon signed rank test: *P*< 1.7e-3), OV (two-sided Wilcoxon signed rank test: *P*< 6.0e-3) and UCEC (two-sided Wilcoxon signed rank test: *P*< 8.4e-4). Similarly, the mRNA + clinical models in COAD (two-sided Wilcoxon signed rank test: *P*< 5.2e-3), LUSC (two-sided Wilcoxon signed rank test: *P*< 1.4e-2), UCEC (two-sided Wilcoxon signed rank test: *P*< 6.5e-3) and OV (two-sided Wilcoxon signed rank test: *P*< 3.1e-3) showed statistically significant increased C-index than their respective molecular-data-only models, which suggested the microRNA and mRNA signatures were more ideal as non-independent prognosis factors in clinical outcomes. Similar results were previously observed in Pan cancer project studies[3, 44]. However, in contrast, few lncRNA/DNA methylation + clinical models were found with degraded performance compared with their respective individual molecular models (Table E in S1 File), including the DNA methylation + clinical models in LUSC and UCEC (two-sided Wilcoxon signed rank test, LUSC: DNA methylation + clinical: *P*< 7.0e-3; UCEC: DNA methylation + clinical: *P*< 2.7e-2), and lncRNA+ clinical models in BRCA and LUSC (two-sided Wilcoxon signed rank test, BRCA: lnc + clinical: *P*< 1.5e-2; LUSC: lnc + clinical: *P*< 3.4e-2), which suggested the two types of molecular approaches can be regarded as higher-level assemblies and act as more robust prognosis factors independent of clinical variables. In addition, we examined the effects of clinical variables on double and triple combination molecular groups in clinical models (Supplementary Methods in S1 File).

## Discussion

In this study, we proposed an IDFO approach to systematically evaluate the prognostic power of diverse molecular data and compared their relative importance across five TCGA cancer types. Importantly, we achieved good stratification of the IDFO approach in most profiling models. Across the five TCGA cancer cohorts, lncRNA illustrated as the best prognostic predictor (‘test sets’) in four cancer types, followed by DNA methylation, then jointly by mRNA and microRNA, the results suggested that the lncRNAs and DNA methylation may potentially play considerable roles in prognosis process. Notably, some of the optimal lncRNA predictors have been well verified in literature suggesting the effectiveness of our analyses in identifying prognosis-relevant markers. Through integrated modeling of multi-type molecular data, we found 80% of the multi-type molecular data showed similar predictive performance to the single-type molecular data, except for lncRNA + DNA methylation in two cancer types of OV and UCEC, suggesting the information content of integrated multi-type molecular data might largely be redundant in terms of survival risk stratification. Moreover, our external validation of IDFO predictors associated with clinical variables in traditional survival analysis not only confirmed the reliability of most IDFO predictors on both dichotomized and censored survival prediction, but also showed a high robustness of lncRNA and DNA methylation signatures as prognosis factors independent of traditional clinical variables. Importantly, similar results had previously been observed in other biomarker identification approaches using Cox models [3, 45]. These results and methods may have specific reference to subsequent related studies.

Currently, only few molecular based markers have been established in clinical practice, as strategies to identify optimal candidate signatures remain a challenge. Although our efforts provided a basis for evaluating patient survival prediction with a systematic model framework, some informative markers may be inevitably missed owing to the multi-co-linearity of high-throughput data and the intra-tumor heterogeneities. Therefore, one important future direction is to develop data-specific approaches to screen out feature panels with more complementary information among diverse high-throughput platforms. Besides, it should be noted that the accuracies of prediction model in microRNA testing cohorts are still limited. For example, only limited microRNAs were available for models owing to tissue-specificity and low dimensionality. On the other hand, recent studies have suggested a nonlinear relationship between microRNA expression and clinical outcomes [46–48], which imply that further studies could assess some nonparametric algorithms on microRNA prognostic analysis.

As is well known, cancer prognosis is likely caused by a series of factors, for example, clinical variables, genetic mutations, and aberrant gene expression. At present, research on the translation of biological data into clinical utility is still limited; therefore, our study has attempted to start the process of bridging this gap. However, as high-throughput technology continues to improve and therapies become increasingly target-specific, more potential markers will inevitably be identified in tandem and will play greater roles in prognostic utility. The integration analysis of diverse molecular profiles provides opportunities to more incorporated practice of clinical oncology.

## Conclusion

In conclusion, we present a prognostic modeling pipeline to specifically evaluate the prognostic power of the lncRNA, mRNA, DNA methylation, and microRNA across five TCGA cancers. Our study determined that lncRNA illustrated the best prognostic performance compared to the three molecular data analysis in four cancer types, followed by DNA methylation, mRNA, and microRNA. Moreover, through integrated modeling of these diverse molecular data, we found 80% of the combined molecular models showed redundancy except for lncRNA + DNA methylation group in two cancers (OV and UCEC). Survival analysis on the IDFO-predictors confirmed the efficacy of our method in identifying prognosis-related markers which may have clinical utility that could be applied to other related studies.

## Supporting Information

S1 File Supplementary files. Model performance with different threshold of feature numbers across four molecular data (Figure A). K-M curves and bar-plot of lncRNA predictors confirmed in literature (Figure B). Overview of tumor samples in four molecular data profiles across five TCGA cancers (Table A). Model performance of diverse molecular data in five TCGA cancers (Table B). The test set accuracies of the 20 integrated molecular models (Table C). Survival analysis of IDFO predictors in five cancers (Table D). Comparison of the prognostic power of molecular data associate with additional clinical variables using clinical models (Table E). List of 22 IDFO—lncRNAs confirmed in literature (Table F). Supplementary Methods.
(DOC)
