# Correction: Comparison of the Prognostic Utility of Diverse Molecular Data among lncRNA, DNA Methylation, microRNA and mRNA across Five Human Cancers

**DOI:** 10.1371/journal.pone.0152631

**Published:** 2016-03-24

**Authors:** Li Xu, Liang Fengji, Liu Changning, Zhang Liangcai, Li Yinghui, Li Yu, Chen Shanguang, Xiong Jianghui

The Funding information is missing from the published article. The corrected funding information is as follows:

This work was supported by the National Instrumentation Program (http://www.most.gov.cn/) grant 2013YQ190467 awarded to XJH, by the Chinese Scientific and Technological Major Special Project (2012ZX09301003-002-003) awarded to XJH, by the Natural Science Foundation of China (http://www.nsfc.gov.cn/) 91129708 awarded to XJH and by grants from the State Key Lab of Space Medicine Fundamentals and Application (internal fund) SMFA09A07, SMFA10A03, andSMFA13A04 award to XJH. The funders had no role in study design, data collection and analysis, decision to publish, or preparation of the manuscript.

S1 File contains errors. The correct S1 File and its caption can be viewed below.

## Supporting Information

S1 File Supplementary files. Model performance with different threshold of feature numbers across four molecular data (Figure A). K-M curves and bar-plot of lncRNA predictors confirmed in literature (Figure B). Overview of tumor samples in four molecular data profiles across five TCGA cancers (Table A). Model performance of diverse molecular data in five TCGA cancers (Table B). The test set accuracies of the 20 integrated molecular models (Table C). Survival analysis of IDFO predictors in five cancers (Table D). Comparison of the prognostic power of molecular data associate with additional clinical variables using clinical models (Table E). List of 22 IDFO—lncRNAs confirmed in literature (Table F). Supplementary Methods.
(DOC)
