# Supplementary material for: Comparison of the Prognostic Utility of the Diverse Molecular Data among lncRNA, DNA Methylation, microRNA, and mRNA across Five Human Cancers
Source: PLoS One. 2015 Nov 25;10(11):e0142433. doi: 10.1371/journal.pone.0142433 (PMC4659652; doi:10.1371/journal.pone.0142433)
Supplement: S1 File — Model performance with different threshold of feature numbers across four molecular data (Figure A). K-M curves and bar-plot of lncRNA predictors confirmed in literature (Figure B). Overview of tumor samples in four molecular data profiles across five TCGA cancers (Table A). Model performance of diverse molecular data in five TCGA cancers (Table B). The test set accuracies of the 20 integrated molecular models (Table C). Survival analysis of IDFO predictors in five cancers (Table D). Comparison of the prognostic power of molecular data associate with additional clinical variables using clinical models (Table E). List of 22 IDFO—lncRNAs confirmed in literature (Table F). Supplementary Methods. (DOC) [file pone.0142433.s001.doc]

**S1 File_Supporting Information**

**Comparison of the Prognostic Utility of Diverse Molecular Data Among lncRNA, DNA methylation, microRNA and mRNA Across Five Human Cancers**

Li Xu^1,2,3^, Liang Fengji^2^, Liu Changning^4^, Zhang Liangcai^5^, Li Yinghui^1,2^, Li Yu^1^, Chen shanguang^1,3,*^, Xiong Jianghui^2,*^

^1^ School of life science and biotechnology, Harbin Institute of Technology, Harbin, Hei Longjiang Province, China

^2^ State Key Laboratory of Space Medicine Fundamentals and Application, Space Institute of Southern China, China Astronaut Research and Training Center, Beijing, China

^3^ National Key Laboratory of Human Factors Engineering, China Astronaut Research and Training Center, Beijing, China

^4^ Xishuangbanna Tropical Botanical Garden, Chinese Academy of Sciences, Yunnan 666303, China

^5^ Department of statistics, Rice University, 6100 main street, Houston TX77005, USA

**Contents**

**Figure A 2**

**Figure B 3**

**Table A 4**

**Table B 5**

**Table C 6**

**Table D 7**

**Table E 9**

**Table F 10**

**Supplementary Methods 11**

**Supplementary References 14**


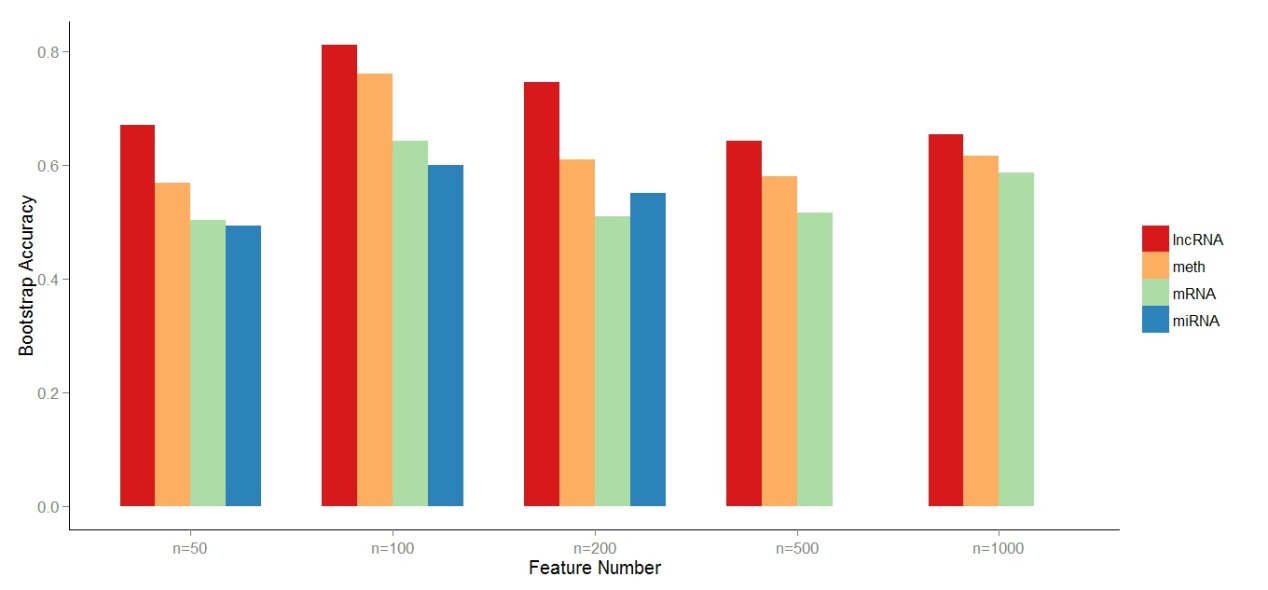


**Figure A. Model performance with different threshold of feature numbers across four molecular data**. Here, n is the number of selected features for modeling. As you noticed, after n=500, the number of microRNAs is insufficient owning to sparse expression and low dimension and was excluded for further modeling. n=100 seems to be the optimal number with highest average accuracies.


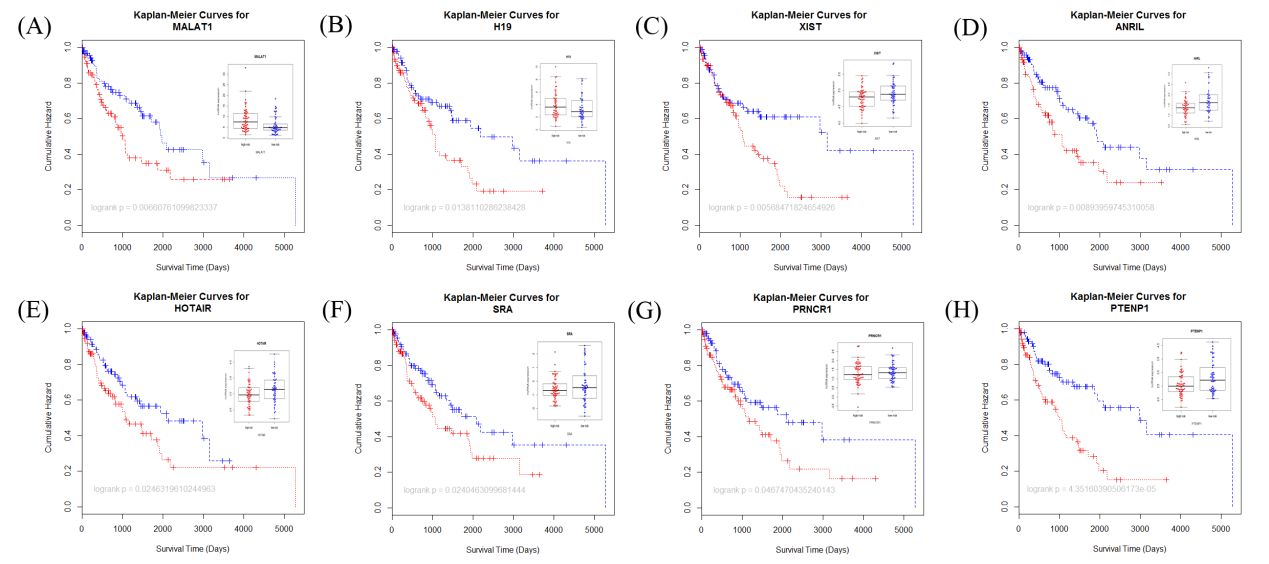


**Figure B.K-M curves and bar-plot of lncRNA predictors confirmed in literature.** The uni-variate proportional hazard model was trained using (A) MALAT1 in LUSC, (B) H19 in OV, (C) XIST in UCEC, (D) ANRIL in BRCA, (E) HOTAIR in LUSC, (F) SRA in OV, (G) PRNCR1 in BRCA, (H) PTENP1 in LUSC. Barplot were generated to describe the expression difference of each predictor among good and bad prognosis patients grouped by Kaplan-Meier Analysis. Red represents bad prognosis patients, blue represents good prognosis patients. The difference in outcome of each predictor was tested using Kaplan-Meier survival analysis (log rank test).

**Table A. Overview of tumor samples in four m**olecular data profiles across five TCGA cancers

| Tumor | Overall Survival | DNAmethy | mRNA | microRNA | lncRNA | Total size |
| --- | --- | --- | --- | --- | --- | --- |
| breast invasive carcinoma  (BRCA) | 869 | 250 | 571 | 848 | 571 | 1098 |
| Colon adenocarcinoma  (COAD) | 423 | 255 | 192 | 187 | 192 | 461 |
| lung squamous cell carcinoma  (LUSC) | 327 | 142 | 220 | 134 | 220 | 504 |
| uterine Corpus Endometrioid Carcinoma (UCEC) | 576 | 329 | 369 | 438 | 369 | 586 |
| serous cystadeno carcinoma  (OV) | 451 | 512 | 263 | 472 | 263 | 548 |

Overall Survival = Total number of patients with non-NA survival time and living status, DNAmethy from the IlluminaInfinium Human DNA Methylation 27K,450K; lncRNA, mRNA from the HiseqV2: Illumina HiSeq 2000 RNA Sequencing V2; microRNA from the Illumina Genome Analyzer/HiSeq 2000 microRNA sequencing platform and Agilent 8 × 15K Human microRNA-specific microarray platform.

**Table B**. Model performance of diverse molecular data in five TCGA cancers

|  | **Molecular data** | **PFS** | **LASSO** | **SVM-RFE** | **RF-IS** | **Monte Carlo simulation** |
| --- | --- | --- | --- | --- | --- | --- |
| **LUSC** | **mRNA** | 0.81 | 0.75 | 0.78 | 0.77 | 0.57 |
|  | **lncRNA** | 0.81 | 0.77 | 0.77 | 0.71 | 0.51 |
|  | **methylation** | 0.93 | 0.91 | 0.96 | 0.89 | 0.63 |
|  | **microRNA** | 0.77 | 0.79 | 0.74 | 0.7 | 0.55 |
| **BRCA** | **mRNA** | 0.77 | 0.71 | 0.75 | 0.73 | 0.54 |
|  | **lncRNA** | 0.83 | 0.81 | 0.77 | 0.81 | 0.59 |
|  | **methylation** | 0.86 | 0.83 | 0.81 | 0.77 | 0.62 |
|  | **microRNA** | 0.68 | 0.69 | 0.67 | 0.68 | 0.55 |
| **COAD** | **mRNA** | 0.92 | 0.87 | 0.91 | 0.85 | 0.61 |
|  | **lncRNA** | 0.95 | 0.94 | 0.92 | 0.88 | 0.67 |
|  | **methylation** | 0.97 | 0.88 | 0.94 | 0.96 | 0.66 |
|  | **microRNA** | 0.79 | 0.84 | 0.81 | 0.77 | 0.63 |
| **OV** | **mRNA** | 0.74 | 0.71 | 0.78 | 0.75 | 0.55 |
|  | **lncRNA** | 0.83 | 0.81 | 0.77 | 0.79 | 0.63 |
|  | **methylation** | 0.78 | 0.82 | 0.79 | 0.81 | 0.61 |
|  | **microRNA** | 0.73 | 0.75 | 0.74 | 0.68 | 0.57 |
| **UCEC** | **mRNA** | 0.86 | 0.79 | 0.85 | 0.8 | 0.62 |
|  | **lncRNA** | 0.94 | 0.88 | 0.93 | 0.81 | 0.62 |
|  | **methylation** | 0.95 | 0.91 | 0.97 | 0.92 | 0.64 |
|  | **microRNA** | 0.71 | 0.83 | 0.81 | 0.7 | 0.59 |

For each data type, we listed the best predictive model of all four feature selection strategies: PFS, LASSO, SVM-RFE, RF-IS in combination with Monte Carlo simulation method.

**Table C**. The test set accuracies of the 20 integrated molecular models

|  | mr+lnr | mr+meth | lnr+meth | mr+lnr+meth |
| --- | --- | --- | --- | --- |
| BRCA | 0.704 | 0.677 | 0.751 | 0.725 |
| COAD | 0.677 | 0.751 | 0.725 | 0.704 |
| LUSC | 0.764 | 0.725 | 0.745 | 0.752 |
| OV | 0.725 | 0.82 | 0.879 | 0.751 |
| UCEC | 0.726 | 0.847 | 0.851 | 0.798 |

The models including the information of 15 double-combination groups and 5 triple-combination groups. For each type of cancer, we have collect the number of overlap samples occured in all three molecular data profiles (mRNA, lncRNA and DNA methylation), including BRCA (n= 178), COAD (n= 161), LUSC (n= 97), OV (n= 145), UCEC (n= 84). Both the double and triple combination models used the overlap samples occurred in all three data profiles in each cancer types, respectively. Lnr = lncRNA, mr = mRNA, meth = DNA methylation

**Table D**. Survival analysis of IDFO predictors in five cancers

| Tumor | Molecular | Likelihood ratio test *p* | Wald's test *p* | ^Log rank test *p* | Likelihood ratio | *Hazard Ratio (mean) | Survival Rates (low risk vs high risk) |
| --- | --- | --- | --- | --- | --- | --- | --- |
| BRCA | lncRNA | 8.76E-05 | 2.74E-05 | 1.76E-05 | 2.38E+01 | 1.21 | 0.92 vs 0.65 |
|  | DNA methylation | 0.009287 | 0.0001 | 0.004669 | 34.48 | 1.22 | 0.99 vs 0.68 |
|  | microRNA | 0.000076 | 0.00434 | 0.008759 | 69.77 | 1.08 | 1 vs 0.16 |
|  | mRNA | 0.0009833 | 0.0002588 | 7.30E-05 | 40.84 | 1.35 | 0.89 vs 0.74 |
| COAD | lncRNA | 4.28E-07 | 0.0348 | 1.33E-06 | 65.94 | 4.78 | 0.91 vs 0.45 |
|  | DNA methylation | 1.38E-07 | 1.00E-08 | 3.80E-10 | 1.90E+02 | 1.77 | 0.97 vs 0.56 |
|  | microRNA | 0.08185 | 0.1233 | 0.1122 | 8.28 | 0.83 | 0.8 vs 0.51 |
|  | mRNA | 2.00E-06 | 1.20E-04 | 8.04E-10 | 2.01E+01 | 2.34 | 0.99 vs 0.37 |
| LUSC | lncRNA | 4.41E-08 | 1.08E-05 | 3.70E-06 | 8.78E+01 | 2.78 | 0.65 vs 0.17 |
|  | DNA methylation | 0.0002266 | 2.76E-05 | 2.54E-06 | 27.93 | 6.91 | 0.99 vs 0.21 |
|  | microRNA | 0.4391 | 0.415 | 0.4014 | 2.71E+00 | 1.56 | 0.41 vs 0.35 |
|  | mRNA | 0.008216 | 4.48E-03 | 1.03E-02 | 38.4 | 2.32 | 0.68 vs 0.21 |
| OV | lncRNA | 9.02E-12 | 6.10E-09 | 1.21E-13 | 1.34E+02 | 2.07 | 0.58 vs 0.09 |
|  | DNA methylation | 0 | 7.71E-13 | 0 | 264.7 | 1.23 | 0.54 vs 0.07 |
|  | microRNA | 0.005956 | 0.009667 | 0.009273 | 14.46 | 0.99 | 0.32 vs 0.24 |
|  | mRNA | 0.0001538 | 0.003332 | 6.58E-05 | 109 | 1.17 | 0.48 vs 0.07 |
| UCEC | lncRNA | 1.97E-11 | 0.5074 | 1.67E-09 | 124.7 | 3.17 | 0.92 vs 0.19 |
|  | DNA methylation | 1.57E-08 | 0.9982 | 0.007316 | 160.6 | 1.61 | 0.99 vs 0.4 |
|  | microRNA | 2.28E-07 | 1.20E-06 | 1.59E-09 | 4.39E+01 | 0.96 | 0.77 vs 0.28 |
|  | mRNA | 0.002289 | 0 | 0.00E+00 | 110.2 | 1.08 | 0.91 vs 0.2 |

The p-value for all two overall tests (likelihood, Wald) was observed for the significant of models with usage variables. *HR (mean) indicated the variables to be related to survival, ^logrank is for testing the difference of two risk groups in survival.

**Table E. Comparison of the prognostic power of molecular data associate with additional clinical variables using clinical models.**

|  | | BRCA | COAD | LUSC | OV | UCEC |
| --- | --- | --- | --- | --- | --- | --- |
| lncRNA | lncRNA (c index) | 0.57 - 0.64 | 0.51 - 0.56 | 0.53 - 0.62 | 0.55 - 0.61 | 0.57 - 0.63 |
|  | lncRNA+clinical (c index) | 0.52 - 0.58 | 0.53 - 0.59 | 0.46 - 0.54 | 0.57 - 0. 63 | 0.55 - 0.66 |
|  | *P value | ↓0.015 | ↑0.483 | ↓0.034 | ↑0.692 | ↑0.333 |
| mRNA | mRNA (c index) | 0.51 - 0.55 | 0.53 - 0.62 | 0.45 - 0.56 | 0.52 - 0.58 | 0.47 - 0.56 |
|  | mRNA+clinical (c index) | 0.54 - 0.56 | 0.60 - 0.64 | 0.62 - 0.70 | 0.55 - 0.68 | 0.61 - 0.74 |
|  | *P value | ↑0.194 | ↑0.0052 | ↑0.014 | ↑0.0031 | ↑0.0065 |
| DNAmethy | DNA methy (c index) | 0.52 - 0.57 | 0.54 - 0.62 | 0.54 - 0.61 | 0.51 - 0.56 | 0.63 - 0.67 |
|  | DNAmethy+clinical (c index) | 0.51 - 0.59 | 0.56 - 0.65 | 0.45 - 0.52 | 0.52 - 0.58 | 0.58 - 0.61 |
|  | *P value | ↑0.371 | ↑0.102 | ↓0.007 | ↑0.309 | ↓0.027 |
| microRNA | microRNA (c index) | 0.51 - 0.57 | 0.53 - 0.58 | 0.48 - 0.55 | 0.48 - 0.53 | 0.59 - 0.62 |
|  | microRNA+clinical (c index) | 0.67 - 0.74 | 0.52 - 0.61 | 0.60 - 0.71 | 0.64 - 0.77 | 0.60 - 0.69 |
|  | *P value | ↑0.0021 | ↑0.275 | ↑0.0017 | ↑0.006 | ↑0.00084 |

C index = concordance.index, a C-index = 1 indicated perfect overall survival prediction and a C-index = 0.5 indicated as good as a random guess

↑indicated an improved performance of molecular models with additional clinical variables

↓indicated an decreased performance of molecular models with additional clinical variables

*P values were calculated to compared the performance (C- index) of each molecular prognostic model with and without clinical variables using two-sided Wilcoxon signed rank test in each cancer types, respectively.

**Table F. List of 22 IDFO - lncRNAs confirmed in literature**

| Ensembl ID | lncRNA gene name | Student's t-test *P* value | Functional annotation |
| --- | --- | --- | --- |
| ENSG00000240498 | *ANRILb* | 0.0089 | Prostate cancer |
| ENSG00000226950 | *DANCR* | 3.03 × 10^−8^ | Development |
| ENSG00000235947 | *EGOT* | 4.8 × 10^−5^ | Development |
| ENSG00000234741 | *GAS5* | 1.97 × 10^−6^ | Breast cancer |
| ENSG00000130600 | *H19* | 0.013 | Multiple cancers |
| ENSG00000228630 | *HOTAIR* | 0.024 | Multiple cancers |
| ENSG00000243766 | *HOTTIP* | 1.5 × 10^−6^ | Development |
| ENSG00000251164 | *HULC* | 0.0311 | Multiple cancers |
| ENSG00000258492 | *KCNQ1OT1* | 0.0103 | Colon cancer |
| ENSG00000251562 | *MALAT1* | 0.006 | Multiple cancers |
| ENSG00000214548 | *MEG3* | 3.92 × 10^−5^ | Multiple cancers |
| ENSG00000245532 | *NEAT1* | 0.00642 | Nuclear speckle |
| ENSG00000225937 | *PCA3* | 9.50 × 10^−3^ | Multiple cancers |
| ENSG00000253438 | *PCAT1* | 1.12 × 10^−7^ | Prostate cancer |
| ENSG00000227418 | *PCGEM1* | 4.49 × 10^−6^ | Prostate cancer |
| ENSG00000238115 | *PRINS* | 1.37 × 10^−9^ | Colon cancer |
| ENST00000519282 | PRNCR1 | 0.046 | Multiple cancers |
| ENST00000447117 | PTENP1 | 4.35× 10^−5^ | lung cancer |
| ENSG00000249859 | *PVT1* | 4.18 × 10^−4^ | Multiple cancers |
|  | *SRA* | 0.024 | lung cancer |
| ENSG00000214049 | *UCA1* | 2.11 × 10^−2^ | Bladder cancer |
| ENST00000602863 | *XIST* | 0.0056 | Uterine cancer |

***** Student's t-test *P* value was adopted to test the difference between two outcome groups classified by IDFO approach.

**Supplementary Methods**

**Data Sources**

In this study, a total of 3198 publicly available tumor profiles were downloaded from The Cancer Genome Atlas (TCGA) project, all tumor samples were collected and processed through the TCGA Biospecimens Core Resource at the International Genomics Consortium, which have been deposited at the Data Coordinating Center of TGCA. Both the clinical data and the molecular data (including DNA methylation, mRNA, lncRNA and microRNA) were obtained from TCGA Data Portal (https://tcga-data.nci.nih.gov/tcga/). The TCGA Data Primer provides an in depth description of TCGA data enterprise including data type and sources (including data levels), the assessment of the data, and the description of the ways to aggregate multi-omic data from the website ([http://cancergenome.nih.gov](http://cancergenome.nih.gov/)).

**Classification algorithms:**

As manuscript mentioned, we built a computational pipeline based on 5 different classification algorithms including: support vector machine (SVM), k-nearest neighbors (KNN), logistic regression (LR), RandomForest (RF) and NaiveBayes (NB).

KNN is one of the simplest machines learning algorithms. In KNN classification, samples are randomly assigned to the class, then select its k nearest neighbors (k is a positive integer) by a majority vote of its neighbors. Although the method is simple and traditional, KNN is often shown good performance comparable to other algorithms.

NaiveBayes (NB) uses the Bayes rule to compute the prior probability of a class of variables. One advantage of NB is that the feature distributions save large amount of space and time in calculation, which solves the problem known as the “curse of dimensionality”. Another advantage is that NB does not need to accurately estimate the absolute accuracy of each class because the classification outcome is determined by the relative probabilities of all classes. We used the R package ‘e1071’ to build the NB classifiers.

Logistic regression (LR) is a generalized linear model for Gaussian/binomial regression. LR has been commonly used to model the outcomes of diverse diseases and data profiles. In this study, we used the R package (base), fitting generalized linear models ‘glm’, to build logistic regression classifiers with standard parameters.

SVM has shown incredible promising performance by providing nonlinear boundaries in a transformed, large version of the feature space. Comparing with other popular algorithms, SVM could achieve higher recognition accuracies. We used the R package ‘e1071’ and ‘PenaltySVM’ to build the SVM classifiers.

The RandomForest (RF) method utilized many individual decision trees to build regression or classification. For each of the tree is performed on random selected samples from the training set. Every RF model in our study was composed of 1000 decision trees using R package (‘caret’ and ‘randomForest’).

**Prioritization-eliminated feature selection (PFS)**

The PFS algorithm was our initially proposed strategy for feature selection, which based on the PRP top-ranked feature sets. It was combined by model building classification algorithms (as above mentioned). Each of the classifier was trained by the low-to-top PRP score ordered feature sets and then iteratively eliminated one number of the least important features with 10000 times random splitting 2/3 training and 1/3 testing. Finally, we calculated the average bootstrap accuracy for each group of feature sets and choose the highest accuracy feature sets among five classifiers as the optimal PFS features.

**Selected numbers in PRP ranks**

We selected top 100 signatures for the PRP calculating. These numbers are chosen because we tried four different threshold of selected numbers including n in 50,100,200,500,1000, and found top 100 could represent the first significant minimums with near-best performance in most molecular data sets (S1 Fig.).

**Calculation of C-index**

We applied the models to a ‘survcomp’ function to calculate the C-index using R package “survcomp”. For each group of feature set, we repeated 1000 times re-sampling to generate 1000 C-indexes. Then the performance of two feature groups was compared based on respective two C index vectors using the Wilcoxon signed rank test to calculate the *P* value (P value above 0.05 as the significance cutoff).

**Clinical TCGA data**

We analyzed DNA methylation and RNA expression (lncRNA, mRNA, microRNA) in combination with clinical data (survival information) from the public TCGA data portal (<https://tcga-data.nci.nih.gov/tcga/>).  We restricted our analysis to patients for which DNA methylation, RNA expression and survival data were both available. The results published here are in whole or part based upon data generated by The Cancer Genome Atlas pilot project established by the NCI and NHGRI. Information about TCGA and the investigators and institutions that constitute the TCGA research network can be found at <http://cancergenome.nih.gov/>.

**Location sources of lncRNA transcripts**

The version of lncRNA location is commonly spread in multiple websites; we assembled the lncRNA positional information from the following databases:

1. **GENCODE v17 (7,346)**
2. **LNCipedia v2.1 (19,324)**
3. **Noncoder v3 (14,382)**
4. **Ensembl (2,275)**
5. **NCBI (4,866)**
6. **UCSC (4,029)**
7. **Agilent G3 v2 (10,796)**

However, after checking the overlaps of different version of lncRNA localizations, we found the different is too large to integration analysis. To avoid ambiguous transcripts mapping, we merged the overlapping lncRNA transcripts occurred in both UCSC and Ensembl into a single candidate lncRNA with a total number of 4560. We calculated both RPKM (read per kilobase of exon per million mapped reads) and TPM value for the expression of lncRNA transcripts, and choose RPKM for the final expression.

**Literature retrieval of IDFO-lncRNA predictors**

NCBI-PubMed retrieval was applied to a full list of 157 lncRNAs appeared in best predictor sets. Encouragingly, 22 popular recognized lncRNAs were identified in our study (Table F). The metastasis associated lung adenocarcinoma transcript 1 (*MALAT1*) as a widely recognized lncRNA, was found to be associated with high metastatic potential and poor patient prognosis in NSCLC, which regulate the level of phosphorylated splicing factors, and thereby affecting alternative splicing in the cell. In our study, MALAT1 show significant over expression in bad prognosis patients in LUSC, but it was also included in COAD best predictor sets (S2A Fig.). H19 as a non-coding tumor indicator was found with loss of imprinting and deregulated in ovarian cancer. It was involved in our OV lncRNA predictor sets (S2B Fig.), which reinforces the role of MALAT1 and H19 as an oncogenic lncRNA, and suggests the MALAT1 regulates poor patient prognosis through early time of lung cancer. Besides, we also found the X-inactive-speciﬁc transcript (XIST) and the antisense non-coding RNA in the INK4 locus (ANRIL) in recent publications (S2C-2D Fig.). ANRIL as an activator of PRC1 and PRC2 were found over-expressed in prostate cancer, these phenomenon obtained in our study suggest ANRIL might play a potential role in pathogenesis of breast cancer prognosis as well. Meanwhile, several other tumor associated lncRNAs (HOTAIR, sra, prncr1, ptenp1) were found with low expression in bad prognosis group and with high expression in good prognosis group in respective cancers (S2E-2H Fig.).The validate of those predictors is important for determining the stability of our method. Also, these results suggest the utilization of our approach could identify trustable/comprehensively prognosis associated lncRNAs, and we guess the newly identified lncRNAs in isolation or as composite markers may be crucial to clinical practice.

**Assessment of the prognostic power of integrated molecular data with clinical variables in traditional clinical models**

To assess the effects of clinical variables on double and triple combination groups in traditional clinical models (Cox regression), we trained the clinical models using IDFO selected optimal feature sets from double and triple combination groups in each type of cancers, respectively. For each combination group set, we repeated 1000 times bootstrap re-sampling to generate 1000 C-indexes and then used the Wilcoxon signed rank test to calculate the *P* values. We found that the prognostic performance of integrated molecular datasets with clinical variables strongly depended on the cancer type. For example, in LUSC and BRCA, the combination molecular + clinical models resulted in improved performance compare to those molecular-data-only models, including: mr+ lnc+clinical in LUSC (two-sided Wilcoxon signed rank test: *P*< 1.1e-2), mr+DNAm+clinical in LUSC (two-sided Wilcoxon signed rank test: *P*< 3.2e-2), mr+DNAm+clinical in BRCA (two-sided Wilcoxon signed rank test: *P*< 4.7e-4), mr+DNAm+lnc+clinical in BRCA (two-sided Wilcoxon signed rank test: *P*< 8.0e-3). Moreover, in COAD , UCEC and OV, most of the integrated molecular data showed similar predictive performance with additional clinical variables, except for the following data sets: mr+DNAm+lnc in COAD (two-sided Wilcoxon signed rank test: *P*< 6.9e-5); mr+lnc in COAD (two-sided Wilcoxon signed rank test: *P*< 2.1e-3), DNAm+mr in UCEC; mr+DNAm+lnc in OV (two-sided Wilcoxon signed rank test: *P*< 4.4e-2), mRNA+lncRNA+DNAmethy in OV (two-sided Wilcoxon signed rank test: *P*< 9.1e-4). In general, the trend observed with clinical models was somewhat similar to those observed using the IDFO approach.

**Supplementary Reference**

1. Laner T, Schulz WA, Engers R, Muller M, Florl AR. Hypomethylation of the XIST gene promoter in prostate cancer. Oncology research. 2005;15(5):257-64. Epub 2005/11/03. PubMed PMID: 16261845.

2. Li CH, Chen Y. Targeting long non-coding RNAs in cancers: progress and prospects. The international journal of biochemistry & cell biology. 2013;45(8):1895-910. Epub 2013/06/12. doi: 10.1016/j.biocel.2013.05.030. PubMed PMID: 23748105.

3. Tong X, Gu PC, Xu SZ, Lin XJ. Long non-coding RNA-DANCR in human circulating monocytes: a potential biomarker associated with postmenopausal osteoporosis. Bioscience, biotechnology, and biochemistry. 2015;79(5):732-7. Epub 2015/02/11. doi: 10.1080/09168451.2014.998617. PubMed PMID: 25660720.

4. Yuan SX, Wang J, Yang F, Tao QF, Zhang J, Wang LL, et al. Long noncoding RNA DANCR increases stemness features of hepatocellular carcinoma via de-repression of CTNNB1. Hepatology (Baltimore, Md). 2015. Epub 2015/05/13. doi: 10.1002/hep.27893. PubMed PMID: 25964079.

5. Rose D, Stadler PF. Molecular evolution of the non-coding eosinophil granule ontogeny transcript. Frontiers in genetics. 2011;2:69. Epub 2012/02/04. doi: 10.3389/fgene.2011.00069. PubMed PMID: 22303364; PubMed Central PMCID: PMCPmc3268622.

6. Pickard MR, Mourtada-Maarabouni M, Williams GT. Long non-coding RNA GAS5 regulates apoptosis in prostate cancer cell lines. Biochimica et biophysica acta. 2013;1832(10):1613-23. Epub 2013/05/17. doi: 10.1016/j.bbadis.2013.05.005. PubMed PMID: 23676682.

7. Pickard MR, Williams GT. Regulation of apoptosis by long non-coding RNA GAS5 in breast cancer cells: implications for chemotherapy. Breast cancer research and treatment. 2014;145(2):359-70. Epub 2014/05/03. doi: 10.1007/s10549-014-2974-y. PubMed PMID: 24789445.

8. Zhang Z, Zhu Z, Watabe K, Zhang X, Bai C, Xu M, et al. Negative regulation of lncRNA GAS5 by miR-21. Cell death and differentiation. 2013;20(11):1558-68. Epub 2013/08/13. doi: 10.1038/cdd.2013.110. PubMed PMID: 23933812; PubMed Central PMCID: PMCPmc3792431.

9. Tu ZQ, Li RJ, Mei JZ, Li XH. Down-regulation of long non-coding RNA GAS5 is associated with the prognosis of hepatocellular carcinoma. International journal of clinical and experimental pathology. 2014;7(7):4303-9. Epub 2014/08/15. PubMed PMID: 25120813; PubMed Central PMCID: PMCPmc4129048.

10. Zhang L, Yang F, Yuan JH, Yuan SX, Zhou WP, Huo XS, et al. Epigenetic activation of the MiR-200 family contributes to H19-mediated metastasis suppression in hepatocellular carcinoma. Carcinogenesis. 2013;34(3):577-86. Epub 2012/12/12. doi: 10.1093/carcin/bgs381. PubMed PMID: 23222811.

11. Zhang EB, Han L, Yin DD, Kong R, De W, Chen J. c-Myc-induced, long, noncoding H19 affects cell proliferation and predicts a poor prognosis in patients with gastric cancer. Medical oncology (Northwood, London, England). 2014;31(5):914. Epub 2014/03/29. doi: 10.1007/s12032-014-0914-7. PubMed PMID: 24671855.

12. Wang L, Cai Y, Zhao X, Jia X, Zhang J, Liu J, et al. Down-regulated long non-coding RNA H19 inhibits carcinogenesis of renal cell carcinoma. Neoplasma. 2015;62(3):412-8. Epub 2015/04/14. doi: 10.4149/neo_2015_049. PubMed PMID: 25866221.

13. Yan L, Zhou J, Gao Y, Ghazal S, Lu L, Bellone S, et al. Regulation of tumor cell migration and invasion by the H19/let-7 axis is antagonized by metformin-induced DNA methylation. Oncogene. 2014;0. Epub 2014/08/05. doi: 10.1038/onc.2014.236. PubMed PMID: 25088204.

14. Li H, Yu B, Li J, Su L, Yan M, Zhu Z, et al. Overexpression of lncRNA H19 enhances carcinogenesis and metastasis of gastric cancer. Oncotarget. 2014;5(8):2318-29. Epub 2014/05/09. PubMed PMID: 24810858; PubMed Central PMCID: PMCPmc4039165.

15. Huang L, Liao LM, Liu AW, Wu JB, Cheng XL, Lin JX, et al. Overexpression of long noncoding RNA HOTAIR predicts a poor prognosis in patients with cervical cancer. Archives of gynecology and obstetrics. 2014;290(4):717-23. Epub 2014/04/22. doi: 10.1007/s00404-014-3236-2. PubMed PMID: 24748337.

16. Qiu JJ, Lin YY, Ye LC, Ding JX, Feng WW, Jin HY, et al. Overexpression of long non-coding RNA HOTAIR predicts poor patient prognosis and promotes tumor metastasis in epithelial ovarian cancer. Gynecologic oncology. 2014;134(1):121-8. Epub 2014/03/26. doi: 10.1016/j.ygyno.2014.03.556. PubMed PMID: 24662839.

17. He X, Bao W, Li X, Chen Z, Che Q, Wang H, et al. The long non-coding RNA HOTAIR is upregulated in endometrial carcinoma and correlates with poor prognosis. International journal of molecular medicine. 2014;33(2):325-32. Epub 2013/11/29. doi: 10.3892/ijmm.2013.1570. PubMed PMID: 24285342.

18. Chen FJ, Sun M, Li SQ, Wu QQ, Ji L, Liu ZL, et al. Upregulation of the long non-coding RNA HOTAIR promotes esophageal squamous cell carcinoma metastasis and poor prognosis. Molecular carcinogenesis. 2013;52(11):908-15. Epub 2013/10/24. doi: 10.1002/mc.21944. PubMed PMID: 24151120.

19. Li J, Wang Y, Yu J, Dong R, Qiu H. A high level of circulating HOTAIR is associated with progression and poor prognosis of cervical cancer. Tumour biology : the journal of the International Society for Oncodevelopmental Biology and Medicine. 2015;36(3):1661-5. Epub 2014/11/05. doi: 10.1007/s13277-014-2765-4. PubMed PMID: 25366139.

20. Quagliata L, Matter MS, Piscuoglio S, Arabi L, Ruiz C, Procino A, et al. Long noncoding RNA HOTTIP/HOXA13 expression is associated with disease progression and predicts outcome in hepatocellular carcinoma patients. Hepatology (Baltimore, Md). 2014;59(3):911-23. Epub 2013/10/12. doi: 10.1002/hep.26740. PubMed PMID: 24114970; PubMed Central PMCID: PMCPmc3943759.

21. Xie H, Ma H, Zhou D. Plasma HULC as a promising novel biomarker for the detection of hepatocellular carcinoma. BioMed research international. 2013;2013:136106. Epub 2013/06/14. doi: 10.1155/2013/136106. PubMed PMID: 23762823; PubMed Central PMCID: PMCPmc3674644.

22. Li C, Chen J, Zhang K, Feng B, Wang R, Chen L. Progress and Prospects of Long Noncoding RNAs (lncRNAs) in Hepatocellular Carcinoma. Cellular physiology and biochemistry : international journal of experimental cellular physiology, biochemistry, and pharmacology. 2015;36(2):423-34. Epub 2015/05/15. doi: 10.1159/000430109. PubMed PMID: 25968300.

23. Peng W, Gao W, Feng J. Long noncoding RNA HULC is a novel biomarker of poor prognosis in patients with pancreatic cancer. Medical oncology (Northwood, London, England). 2014;31(12):346. Epub 2014/11/22. doi: 10.1007/s12032-014-0346-4. PubMed PMID: 25412939.

24. Vausort M, Wagner DR, Devaux Y. Long noncoding RNAs in patients with acute myocardial infarction. Circulation research. 2014;115(7):668-77. Epub 2014/07/19. doi: 10.1161/circresaha.115.303836. PubMed PMID: 25035150.

25. Wang J, Su L, Chen X, Li P, Cai Q, Yu B, et al. MALAT1 promotes cell proliferation in gastric cancer by recruiting SF2/ASF. Biomedicine & pharmacotherapy = Biomedecine & pharmacotherapie. 2014;68(5):557-64. Epub 2014/05/27. doi: 10.1016/j.biopha.2014.04.007. PubMed PMID: 24857172.

26. Ji Q, Zhang L, Liu X, Zhou L, Wang W, Han Z, et al. Long non-coding RNA MALAT1 promotes tumour growth and metastasis in colorectal cancer through binding to SFPQ and releasing oncogene PTBP2 from SFPQ/PTBP2 complex. Br J Cancer. 2014;111(4):736-48. Epub 2014/07/16. doi: 10.1038/bjc.2014.383. PubMed PMID: 25025966; PubMed Central PMCID: PMCPmc4134507.

27. Wu XS, Wang XA, Wu WG, Hu YP, Li ML, Ding Q, et al. MALAT1 promotes the proliferation and metastasis of gallbladder cancer cells by activating the ERK/MAPK pathway. Cancer biology & therapy. 2014;15(6):806-14. Epub 2014/03/25. doi: 10.4161/cbt.28584. PubMed PMID: 24658096; PubMed Central PMCID: PMCPmc4049796.

28. Zheng HT, Shi DB, Wang YW, Li XX, Xu Y, Tripathi P, et al. High expression of lncRNA MALAT1 suggests a biomarker of poor prognosis in colorectal cancer. International journal of clinical and experimental pathology. 2014;7(6):3174-81. Epub 2014/07/18. PubMed PMID: 25031737; PubMed Central PMCID: PMCPmc4097248.

29. Shen L, Chen L, Wang Y, Jiang X, Xia H, Zhuang Z. Long noncoding RNA MALAT1 promotes brain metastasis by inducing epithelial-mesenchymal transition in lung cancer. Journal of neuro-oncology. 2015;121(1):101-8. Epub 2014/09/15. doi: 10.1007/s11060-014-1613-0. PubMed PMID: 25217850.

30. Ma KX, Wang HJ, Li XR, Li T, Su G, Yang P, et al. Long noncoding RNA MALAT1 associates with the malignant status and poor prognosis in glioma. Tumour biology : the journal of the International Society for Oncodevelopmental Biology and Medicine. 2015. Epub 2015/01/24. doi: 10.1007/s13277-014-2969-7. PubMed PMID: 25613066.

31. Pang EJ, Yang R, Fu XB, Liu YF. Overexpression of long non-coding RNA MALAT1 is correlated with clinical progression and unfavorable prognosis in pancreatic cancer. Tumour biology : the journal of the International Society for Oncodevelopmental Biology and Medicine. 2015;36(4):2403-7. Epub 2014/12/08. doi: 10.1007/s13277-014-2850-8. PubMed PMID: 25481511.

32. Zhang HM, Yang FQ, Chen SJ, Che J, Zheng JH. Upregulation of long non-coding RNA MALAT1 correlates with tumor progression and poor prognosis in clear cell renal cell carcinoma. Tumour biology : the journal of the International Society for Oncodevelopmental Biology and Medicine. 2015;36(4):2947-55. Epub 2014/12/07. doi: 10.1007/s13277-014-2925-6. PubMed PMID: 25480417.

33. Jia LF, Wei SB, Gan YH, Guo Y, Gong K, Mitchelson K, et al. Expression, regulation and roles of miR-26a and MEG3 in tongue squamous cell carcinoma. International journal of cancer Journal international du cancer. 2014;135(10):2282-93. Epub 2013/12/18. doi: 10.1002/ijc.28667. PubMed PMID: 24343426.

34. Yan J, Guo X, Xia J, Shan T, Gu C, Liang Z, et al. MiR-148a regulates MEG3 in gastric cancer by targeting DNA methyltransferase 1. Medical oncology (Northwood, London, England). 2014;31(3):879. Epub 2014/02/12. doi: 10.1007/s12032-014-0879-6. PubMed PMID: 24515776.

35. Zhuo H, Tang J, Lin Z, Jiang R, Zhang X, Ji J, et al. The aberrant expression of MEG3 regulated by UHRF1 predicts the prognosis of hepatocellular carcinoma. Molecular carcinogenesis. 2015. Epub 2015/02/03. doi: 10.1002/mc.22270. PubMed PMID: 25641194.

36. Sun M, Xia R, Jin F, Xu T, Liu Z, De W, et al. Downregulated long noncoding RNA MEG3 is associated with poor prognosis and promotes cell proliferation in gastric cancer. Tumour biology : the journal of the International Society for Oncodevelopmental Biology and Medicine. 2014;35(2):1065-73. Epub 2013/09/06. doi: 10.1007/s13277-013-1142-z. PubMed PMID: 24006224.

37. Yin DD, Liu ZJ, Zhang E, Kong R, Zhang ZH, Guo RH. Decreased expression of long noncoding RNA MEG3 affects cell proliferation and predicts a poor prognosis in patients with colorectal cancer. Tumour biology : the journal of the International Society for Oncodevelopmental Biology and Medicine. 2015. Epub 2015/02/01. doi: 10.1007/s13277-015-3139-2. PubMed PMID: 25636452.

38. Choudhry H, Albukhari A, Morotti M, Hider S, Moralli D, Smythies J, et al. Tumor hypoxia induces nuclear paraspeckle formation through HIF-2alpha dependent transcriptional activation of NEAT1 leading to cancer cell survival. Oncogene. 2014. Epub 2014/11/25. doi: 10.1038/onc.2014.378. PubMed PMID: 25417700; PubMed Central PMCID: PMCPmc4430310.

39. Wang Y, Liu XJ, Yao XD. Function of PCA3 in prostate tissue and clinical research progress on developing a PCA3 score. Chinese journal of cancer research = Chung-kuo yen cheng yen chiu. 2014;26(4):493-500. Epub 2014/09/19. doi: 10.3978/j.issn.1000-9604.2014.08.08. PubMed PMID: 25232225; PubMed Central PMCID: PMCPmc4153936.

40. Leyten GH, Hessels D, Jannink SA, Smit FP, de Jong H, Cornel EB, et al. Prospective multicentre evaluation of PCA3 and TMPRSS2-ERG gene fusions as diagnostic and prognostic urinary biomarkers for prostate cancer. European urology. 2014;65(3):534-42. Epub 2012/12/04. doi: 10.1016/j.eururo.2012.11.014. PubMed PMID: 23201468.

41. Chevli KK, Duff M, Walter P, Yu C, Capuder B, Elshafei A, et al. Urinary PCA3 as a predictor of prostate cancer in a cohort of 3,073 men undergoing initial prostate biopsy. The Journal of urology. 2014;191(6):1743-8. Epub 2013/12/18. doi: 10.1016/j.juro.2013.12.005. PubMed PMID: 24333241.

42. Soumarova R, Boday A, Krhutova V, Janotova A, Dvorakova M, Jaluvkova E, et al. Prognostic and predictive molecular biological markers in prostate cancer - significance of expression of genes PCA3 and TMPRSS2. Neoplasma. 2015;62(1):114-8. Epub 2015/01/08. PubMed PMID: 25563374.

43. Ferro M, Bruzzese D, Perdona S, Marino A, Mazzarella C, Perruolo G, et al. Prostate Health Index (Phi) and Prostate Cancer Antigen 3 (PCA3) significantly improve prostate cancer detection at initial biopsy in a total PSA range of 2-10 ng/ml. PloS one. 2013;8(7):e67687. Epub 2013/07/19. doi: 10.1371/journal.pone.0067687. PubMed PMID: 23861782; PubMed Central PMCID: PMCPmc3701535.

44. Eldai H, Periyasamy S, Al Qarni S, Al Rodayyan M, Muhammed Mustafa S, Deeb A, et al. Novel genes associated with colorectal cancer are revealed by high resolution cytogenetic analysis in a patient specific manner. PloS one. 2013;8(10):e76251. Epub 2013/11/10. doi: 10.1371/journal.pone.0076251. PubMed PMID: 24204606; PubMed Central PMCID: PMCPmc3813709.

45. Walsh AL, Tuzova AV, Bolton EM, Lynch TH, Perry AS. Long noncoding RNAs and prostate carcinogenesis: the missing 'linc'? Trends in molecular medicine. 2014;20(8):428-36. Epub 2014/05/20. doi: 10.1016/j.molmed.2014.03.005. PubMed PMID: 24836411.

46. Bialkowska-Hobrzanska H, Driman DK, Fletcher R, Harry V, Razvi H. Expression of human telomerase reverse transcriptase, Survivin, DD3 and PCGEM1 messenger RNA in archival prostate carcinoma tissue. The Canadian journal of urology. 2006;13(1):2967-74. Epub 2006/03/07. PubMed PMID: 16515751.

47. Prensner JR, Sahu A, Iyer MK, Malik R, Chandler B, Asangani IA, et al. The IncRNAs PCGEM1 and PRNCR1 are not implicated in castration resistant prostate cancer. Oncotarget. 2014;5(6):1434-8. Epub 2014/04/15. PubMed PMID: 24727738; PubMed Central PMCID: PMCPmc4039221.

48. Szegedi K, Sonkoly E, Nagy N, Nemeth IB, Bata-Csorgo Z, Kemeny L, et al. The anti-apoptotic protein G1P3 is overexpressed in psoriasis and regulated by the non-coding RNA, PRINS. Experimental dermatology. 2010;19(3):269-78. Epub 2010/04/10. doi: 10.1111/j.1600-0625.2010.01066.x. PubMed PMID: 20377629.

49. Sonkoly E, Bata-Csorgo Z, Pivarcsi A, Polyanka H, Kenderessy-Szabo A, Molnar G, et al. Identification and characterization of a novel, psoriasis susceptibility-related noncoding RNA gene, PRINS. The Journal of biological chemistry. 2005;280(25):24159-67. Epub 2005/04/28. doi: 10.1074/jbc.M501704200. PubMed PMID: 15855153.

50. Li L, Sun R, Liang Y, Pan X, Li Z, Bai P, et al. Association between polymorphisms in long non-coding RNA PRNCR1 in 8q24 and risk of colorectal cancer. Journal of experimental & clinical cancer research : CR. 2013;32:104. Epub 2013/12/18. doi: 10.1186/1756-9966-32-104. PubMed PMID: 24330491; PubMed Central PMCID: PMCPmc4029281.

51. Marsit CJ, Zheng S, Aldape K, Hinds PW, Nelson HH, Wiencke JK, et al. PTEN expression in non-small-cell lung cancer: evaluating its relation to tumor characteristics, allelic loss, and epigenetic alteration. Human pathology. 2005;36(7):768-76. Epub 2005/08/09. doi: 10.1016/j.humpath.2005.05.006. PubMed PMID: 16084946.

52. Dong L, Qi P, Xu MD, Ni SJ, Huang D, Xu QH, et al. Circulating CUDR, LSINCT-5 and PTENP1 long noncoding RNAs in sera distinguish patients with gastric cancer from healthy controls. International journal of cancer Journal international du cancer. 2015. Epub 2015/02/20. doi: 10.1002/ijc.29484. PubMed PMID: 25694351.

53. Huang CS, Yu W, Cui H, Wang YJ, Zhang L, Han F, et al. Increased expression of the lncRNA PVT1 is associated with poor prognosis in pancreatic cancer patients. Minerva medica. 2015. Epub 2015/02/11. PubMed PMID: 25668599.

54. Kong R, Zhang EB, Yin DD, You LH, Xu TP, Chen WM, et al. Long noncoding RNA PVT1 indicates a poor prognosis of gastric cancer and promotes cell proliferation through epigenetically regulating p15 and p16. Molecular cancer. 2015;14(1):82. Epub 2015/04/19. doi: 10.1186/s12943-015-0355-8. PubMed PMID: 25890171; PubMed Central PMCID: PMCPmc4399399.

55. Tseng YY, Moriarity BS, Gong W, Akiyama R, Tiwari A, Kawakami H, et al. PVT1 dependence in cancer with MYC copy-number increase. Nature. 2014;512(7512):82-6. Epub 2014/07/22. doi: 10.1038/nature13311. PubMed PMID: 25043044.

56. Yi H, Guo C, Yu X, Gao P, Qian J, Zuo D, et al. Targeting the immunoregulator SRA/CD204 potentiates specific dendritic cell vaccine-induced T-cell response and antitumor immunity. Cancer research. 2011;71(21):6611-20. Epub 2011/09/15. doi: 10.1158/0008-5472.can-11-1801. PubMed PMID: 21914786; PubMed Central PMCID: PMCPmc3213980.

57. Zhou C, Chen H, Han L, Xue F, Wang A, Liang YJ. Screening of genes related to lung cancer caused by smoking with RNA-Seq. European review for medical and pharmacological sciences. 2014;18(1):117-25. Epub 2014/01/24. PubMed PMID: 24452952.

58. Li JY, Ma X, Zhang CB. Overexpression of long non-coding RNA UCA1 predicts a poor prognosis in patients with esophageal squamous cell carcinoma. International journal of clinical and experimental pathology. 2014;7(11):7938-44. Epub 2015/01/01. PubMed PMID: 25550835; PubMed Central PMCID: PMCPmc4270573.

59. Juan-Mateu J, Rodriguez MJ, Nascimento A, Jimenez-Mallebrera C, Gonzalez-Quereda L, Rivas E, et al. Prognostic value of X-chromosome inactivation in symptomatic female carriers of dystrophinopathy. Orphanet journal of rare diseases. 2012;7:82. Epub 2012/10/25. doi: 10.1186/1750-1172-7-82. PubMed PMID: 23092449; PubMed Central PMCID: PMCPmc3492175.

60. Vapnik VN. An overview of statistical learning theory. IEEE transactions on neural networks / a publication of the IEEE Neural Networks Council. 1999;10(5):988-99. Epub 2008/02/07. doi: 10.1109/72.788640. PubMed PMID: 18252602.

61. Wu Y, Ianakiev K, Govindaraju V. Improvements in K-Nearest Neighbor Classification. In: Singh S, Murshed N, Kropatsch W, editors. Advances in Pattern Recognition — ICAPR 2001. Lecture Notes in Computer Science. 2013: Springer Berlin Heidelberg; 2001. p. 224-31.

62. Ribbing J, Nyberg J, Caster O, Jonsson EN. The lasso—a novel method for predictive covariate model building in nonlinear mixed effects models. J Pharmacokinet Pharmacodyn. 2007;34(4):485-517. doi: 10.1007/s10928-007-9057-1.

63. Breiman L. Random Forests. Machine Learning. 2001;45(1):5-32. doi: 10.1023/A:1010933404324.

64. Friedman N, Geiger D, Goldszmidt M. Bayesian Network Classifiers. Machine Learning. 1997;29(2-3):131-63. doi: 10.1023/A:1007465528199.

65. Harrell FE, Jr., Lee KL, Mark DB. Multivariable prognostic models: issues in developing models, evaluating assumptions and adequacy, and measuring and reducing errors. Statistics in medicine. 1996;15(4):361-87. Epub 1996/02/28. doi: 10.1002/(sici)1097-0258(19960229)15:4<361::aid-sim168>3.0.co;2-4. PubMed PMID: 8668867.

66. Lyth DH. Formulations of the ϱ bootstrap. Nuov Cim A. 1971;6(3):438-. doi: 10.1007/BF02728585.

67. Gutschner T, Hammerle M, Eissmann M, Hsu J, Kim Y, Hung G, et al. The noncoding RNA MALAT1 is a critical regulator of the metastasis phenotype of lung cancer cells. Cancer research. 2013;73(3):1180-9. Epub 2012/12/18. doi: 10.1158/0008-5472.can-12-2850. PubMed PMID: 23243023; PubMed Central PMCID: PMCPmc3589741.

68. Zhao W, An Y, Liang Y, Xie XW. Role of HOTAIR long noncoding RNA in metastatic progression of lung cancer. European review for medical and pharmacological sciences. 2014;18(13):1930-6. Epub 2014/07/11. PubMed PMID: 25010625.

69. Lin K, Ma J, Wu R, Zhou C, Lin J. Influence of ovarian endometrioma on expression of steroid receptor RNA activator, estrogen receptors, vascular endothelial growth factor, and thrombospondin 1 in the surrounding ovarian tissues. Reproductive sciences (Thousand Oaks, Calif). 2014;21(2):183-9. Epub 2013/06/12. doi: 10.1177/1933719113492205. PubMed PMID: 23749764; PubMed Central PMCID: PMCPmc3879988.

70. Sacco JJ, Yau TY, Darling S, Patel V, Liu H, Urbe S, et al. The deubiquitylase Ataxin-3 restricts PTEN transcription in lung cancer cells. Oncogene. 2014;33(33):4265-72. Epub 2013/12/03. doi: 10.1038/onc.2013.512. PubMed PMID: 24292675; PubMed Central PMCID: PMCPmc4351423.
